# Defective Base Excision Repair of Oxidative DNA Damage in Vascular Smooth Muscle Cells Promotes Atherosclerosis

**DOI:** 10.1161/CIRCULATIONAHA.117.033249

**Published:** 2018-10-01

**Authors:** Aarti Shah, Kelly Gray, Nichola Figg, Alison Finigan, Lakshi Starks, Martin Bennett

**Affiliations:** Division of Cardiovascular Medicine, University of Cambridge, Addenbrooke’s Centre for Clinical Investigation, Addenbrooke’s Hospital, United Kingdom. Dr Gray is currently at Cardiovascular Safety, AstraZeneca, Cambridge, United Kingdom.

**Keywords:** atherosclerosis, DNA damage, DNA glycosylases, oxidative stress, vascular diseases

## Abstract

**Background:**

Atherosclerotic plaques demonstrate extensive accumulation of oxidative DNA damage, predominantly as 8-oxoguanine (8oxoG) lesions. 8oxoG is repaired by base excision repair enzymes; however, the mechanisms regulating 8oxoG accumulation in vascular smooth muscle cells (VSMCs) and its effects on their function and in atherosclerosis are unknown.

**Methods:**

We studied levels of 8oxoG and its regulatory enzymes in human atherosclerosis, the mechanisms regulating 8oxoG repair and the base excision repair enzyme 8oxoG DNA glycosylase I (OGG1) in VSMCs in vitro, and the effects of reducing 8oxoG in VSMCs in atherosclerosis in ApoE^−/−^ mice.

**Results:**

Human plaque VSMCs showed defective nuclear 8oxoG repair, associated with reduced acetylation of OGG1. OGG1 was a key regulatory enzyme of 8oxoG repair in VSMCs, and its acetylation was crucial to its repair function through regulation of protein stability and expression. p300 and sirtuin 1 were identified as the OGG1 acetyltransferase and deacetylase regulators, respectively, and both proteins interacted with OGG1 and regulated OGG1 acetylation at endogenous levels. However, p300 levels were decreased in human plaque VSMCs and in response to oxidative stress, suggesting that reactive oxygen species–induced regulation of OGG1 acetylation could be caused by reactive oxygen species–induced decrease in p300 expression. We generated mice that express VSMC-restricted OGG1 or an acetylation defective version (SM22α-OGG1 and SM22α-OGG1^K-R^ mice) and crossed them with ApoE^−/−^ mice. We also studied ApoE^−/−^ mice deficient in OGG1 (OGG1^−/−^). OGG1^−/−^ mice showed increased 8oxoG in vivo and increased atherosclerosis, whereas mice expressing VSMC-specific OGG1 but not the acetylation mutant OGG1^K-R^ showed markedly reduced intracellular 8oxoG and reduced atherosclerosis. VSMC OGG1 reduced telomere 8oxoG accumulation, DNA strand breaks, cell death and senescence after oxidant stress, and activation of proinflammatory pathways.

**Conclusions:**

We identify defective 8oxoG base excision repair in human atherosclerotic plaque VSMCs, OGG1 as a major 8oxoG repair enzyme in VSMCs, and p300/sirtuin 1 as major regulators of OGG1 through acetylation/deacetylation. Reducing oxidative damage by rescuing OGG1 activity reduces plaque development, indicating the detrimental effects of 8oxoG on VSMC function.

## Abstract 2

Supplemental Digital Content is available in the text.

**Clinical Perspective**

**What Is New?**

- We demonstrate that human atherosclerosis exhibits increased oxidative DNA damage and defective repair of that damage in vascular smooth muscle cells (VSMCs).
- Defective base excision repair is caused by reduced expression, acetylation, and activity of the enzyme 8-oxoguanine DNA glycosylase in atherosclerosis.
- 8-Oxoguanine DNA glycosylase is a major base excision repair enzyme in VSMCs, the activity and protein stability of which are regulated by acetylation through the p300 acetyltransferase and sirtuin 1 deacetylase enzymes.
- Correcting the base excision repair defect in VSMCs alone markedly reduces plaque formation, indicating that endogenous levels of oxidative DNA damage in VSMCs promote plaque development.

**What Are the Clinical Implications?**

- Oxidative DNA damage accumulates slowly in atherosclerosis and disappears only very slowly when hyperlipidemia is corrected.
- Oxidative DNA damage causes inflammation, cell death, and cell senescence, all of which promote atherogenesis.
- 8-Oxoguanine DNA glycosylase protects VSMCs against oxidative DNA damage, identifying base excision repair as a possible therapeutic target in atherosclerosis.
- Protection against oxidative DNA damage or increased DNA repair is beneficial over and above the standard clinical approach of reducing risk factors for coronary artery disease that promote damage, including hypercholesterolemia, diabetes mellitus, and smoking.

DNA bases are susceptible to oxidation mediated by reactive oxygen species (ROS). The low redox potential of guanine makes it especially vulnerable and leads to a plethora of oxidized guanine products.^1^ 8-Oxoguanine (8oxoG) is the most abundant DNA lesion formed on oxidative exposure, and the presence of 8oxoG is often used as a cellular biomarker to indicate the extent of oxidative stress. 8oxoG is a highly mutagenic miscoding lesion that can lead to G:C to T:A transversion mutations and is widely found in human disease and aging.^2^ However, it is often unclear whether 8oxoG accumulation is just a marker of oxidative stress or has a pathogenetic role in disease.

Base excision repair (BER) is the primary mechanism for repairing 8oxoG. BER involves the concerted effort of several repair proteins that recognize and excise oxidized bases, replacing the damaged moiety with a normal nucleotide and restoring DNA integrity.^3^ BER is a critical process for genomic maintenance, as highlighted by the severe phenotypes of mice deficient in BER function, including premature aging and metabolic defects.^4^ However, except for specific BER gene mutations, evidence of defective BER and whether it contributes to human disease is limited.

Advanced atherosclerotic plaques are characterized by 8oxoG accumulation in vascular smooth muscle cells (VSMCs), macrophages, and endothelial cells.^5,6^ 8oxoG also accumulates in plaques in fat-fed animals but normalizes only slowly on a normal diet.^5^ The persistence of DNA damage can reflect both ongoing damage-inducing stimuli, for example, through ROS, and defects in DNA repair. A number of inherited defects that impair DNA repair are associated with human atherosclerosis or can promote atherosclerosis in animal models (reviewed elsewhere^7^). In contrast, whether the far lower endogenous levels of 8oxoG found in atherosclerosis affect plaque development is not known. A recent study has shown that knockout of the DNA glycosylase 8oxoG DNA glycosylase (OGG1) in macrophages promotes atherosclerosis and that OGG1 transcript expression was reduced in human plaques compared with normal vessels^8^; however, whether the observed decreased OGG1 expression translates into DNA repair defects is unknown.

We show that human atherosclerotic plaque VSMCs have defective 8oxoG BER, associated with decreased expression and acetylation of OGG1. We establish OGG1 as a major 8oxoG repair enzyme in VSMCs and that OGG1 activity in VSMCs is controlled by lysine 338/341 acetylation. We identify p300 and sirtuin 1 (SIRT1) as major acetyltransferase and deacetylase enzymes directly targeting OGG1 and thus regulating 8oxoG BER and 8oxoG content in VSMCs. p300 expression is reduced in plaque VSMCs and by oxidative stress, and reduced formation of the p300-OGG1 complex compromises OGG1 activity and protein stability. Inhibiting endogenous oxidative damage by rescuing VSMC OGG1 markedly reduces atherosclerosis in vivo, an effect that requires OGG1 acetylation. Our findings indicate that human atherosclerosis is characterized by defective 8oxoG BER and that endogenous levels of oxidative DNA damage in VSMCs promote atherosclerosis.

## Methods

The data that support the findings of this study are available within the article, in the Data Supplement, and from the corresponding author on reasonable request.

### Human Atherosclerotic Plaque and Normal Vessels

Human tissue was obtained under informed consent with protocols approved by the Cambridge or Huntingdon Research Ethical Committee. Atherosclerotic plaques and normal aorta were obtained from separate patients undergoing carotid endarterectomy or aortic valve replacement, respectively.

### Experimental Animals

All in vivo experiments followed UK Home Office licensing and were approved by the local animal ethics committee. Transgenic mice were generated as described in the online-only Data Supplement. The global OGG^−/−^ and SM22α-SIRT1^ex4/ex4^ conditional transgenic mouse models were generated as described previously.^9,10^ OGG1^−/−^ mouse embryos were a gift from Christi Walter (University of Texas Health Science Center, Houston, TX).

### Atherosclerosis Protocols

Male and female littermate control ApoE^−/−^, SM22α-OGG1/ApoE^−/−^, and SM22α-OGG1^K-R^/ApoE^−/−^ mice were fed high-fat Western diet (829-100, Special Diet Services, 21% total fat, 0.2% cholesterol, 0% sodium cholate) from 8 to 22 weeks.

### Histological Analysis and Oil Red O Staining of Descending Aorta

Atherosclerosis extent and composition were analyzed as described previously^10^ and in the online-only Data Supplement.

### Cell Culture

Human, rat, and mouse VSMCs were cultured as described previously^11^ and in the online-only Data Supplement.

### Transfections and Virus Infections

Transfections and retrovirus infections were performed as described previously^10^ and in the online-only Data Supplement.

### CRISPR-Mediated Gene Silencing

Gene silencing experiments were performed as described in the online-only Data Supplement.

### Real-Time Polymerase Chain Reaction

Oligonucleotide sequences used are listed in Table I in the online-only Data Supplement.

### Oligonucleotide Incision Assay

8oxoG BER activity in nuclear lysates was determined as described previously^12^ and in the online-only Data Supplement.

### 8oxoG ELISA

8oxoG levels were assessed with an ELISA assay (Abcam) as described in the online-only Data Supplement.

### Intracellular ROS Measurement

Intracellular ROS was measured as described previously^13^ and in the online-only Data Supplement.

### Annexin V/PI Flow Cytometry

Cell death was determined with an apoptosis detection kit (BD BioSciences) as described in the online-only Data Supplement.

### Comet Assay

Comet assay was performed with mouse VSMCs as described previously^11^ and in the online-only Data Supplement.

### Immunofluorescence

Immunofluorescence analysis was performed as described previously^14^ and in the online-only Data Supplement.

### Immunoprecipitation and Western blotting

Immunoprecipitation assays and Western blotting were performed as described previously^11^ and in the online-only Data Supplement.

### Chromatin Immunoprecipitation–Quantitative Polymerase Chain Reaction

Chromatin immunoprecipitation was performed as described in the online-only Data Supplement.

### Statistical Analysis

Sample sizes were selected on the basis of previous experiments that identified significant differences in plaque development in mice. No randomization was applied because all mice used were genetically defined, inbred mice. Blinding was used, and no animals were excluded from analysis. Data shown are mean±SEM. Normality of distribution was determined with D’Agostino-Pearson omnibus normality tests. Statistical significance was determined by 1-way ANOVA followed by Bonferroni posttest when >2 groups were compared and a 2-tailed Student *t* test to compare 1 groups of data using Prism 6.0 (Graph Pad). Differences were considered statistically significant at a value of *P*<0.05.

## Results

### Human Atherosclerotic Plaque VSMCs Show Reduced BER Activity

**Figure 1.**
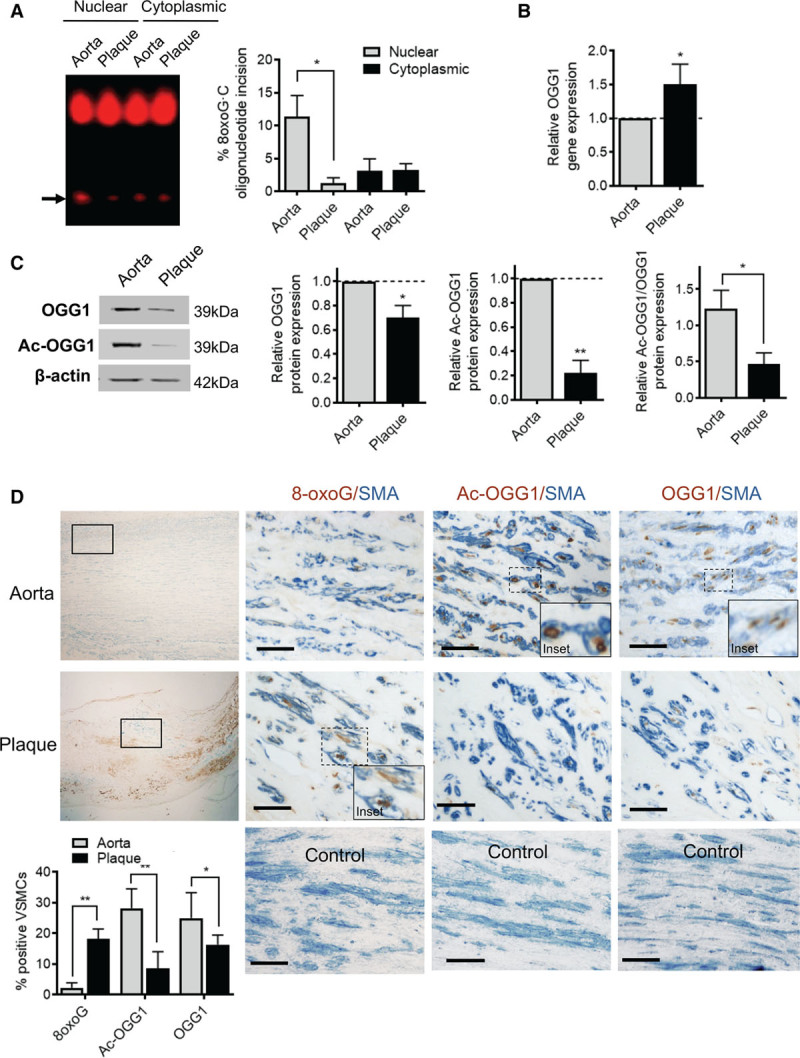
**Human plaque VSMCs show defective nuclear BER activity and reduced acetyl-OGG1 expression. A**, Base excision repair (BER) assay measuring incision of a fluorescently labeled 8-oxoguanine (8oxoG.C) oligonucleotide in nuclear and cytoplasmic fractions of cultured plaque and normal aortic vascular smooth muscle cells (VSMCs). Representative gel (**Left**) and quantification (**Right**) are shown. Incision product is denoted by an arrow (n=4). **B**, Quantitative polymerase chain reaction analysis of 8oxoG DNA glycosylase I (OGG1) expression in cultured plaque and normal aortic VSMCs (n=4). **C**, Western blot of OGG1 and acetylated OGG1 (Ac-OGG1) in plaque and normal aortic VSMCs (n=4). **D**, Immunohistochemistry for 8oxoG, Ac-OGG1, or OGG1 (brown) in sections of human plaques and normal aorta (n=10). Sections are also costained for α-smooth muscle actin (SMA; blue). Insets show high-power views of outlined areas. Negative controls using isotype-matched antibodies and quantification are shown below. Scale bars, 25 µm. All graphical data are mean±SEM. *P<0.05, **P<0.01, Student *t* test.

Previous studies have shown that advanced human atherosclerotic plaques display increased 8oxoG lesions in cells expressing VSMC or macrophage markers compared with normal arteries.^15^ Studies also show that plaques have increased ROS content compared with normal vessels,^16,17^ so increased 8oxoG may be caused by increased oxidative stress within the plaque, defective BER, or both. We therefore examined 8oxoG repair in VSMCs cultured from human carotid plaques or normal aorta from patients matched for age and sex, with cultures matched for passage number. Cells were fractionated into nuclear and cytoplasmic compartments to assay nuclear or mitochondrial BER, respectively (Figure I in the online-only Data Supplement), and 8oxoG repair activity was examined with a fluorescently labeled 8oxoG-containing molecular beacon that can be incised and assayed in real time. Plaque VSMCs showed a marked reduction in nuclear 8oxoG repair activity compared with aortic VSMCs, but cytoplasmic 8oxoG repair activity was similar in both cell types (Figure 1A).

Although 8oxoG repair is mediated by a number of enzymes, knockout studies suggest that OGG1 is a major, nonredundant enzyme responsible for repairing 8oxoG from the bulk of the genome in many tissues^18^ and that activation of alternative pathways cannot compensate for OGG1 deficiency. OGG1 mRNA was increased in human plaque VSMCs 1.5-fold on quantitative polymerase chain reaction (Figure 1B), but plaque VSMCs showed reduced total OGG1 protein expression compared with aortic VSMCs and a marked reduction in acetyl-OGG1 (Ac-OGG1) expression (Figure 1C). To determine whether protein expression in vitro reflects expression in VSMCs in vivo, we examined 8oxoG, Ac-OGG1, and OGG1 expression in human coronary plaques from American Heart Association grade IV lesions compared with normal undiseased aorta, colabeled with α-smooth muscle actin to identify VSMCs. 8oxoG lesions were increased in human plaque VSMCs (Figure 1D). Consistent with the in vitro data, human plaque VSMCs showed a reduced percentage of VSMCs expressing total OGG1 and a markedly reduced percentage of VSMCs expressing Ac-OGG1 versus aortic VSMCs (Figure 1D), suggesting that increased oxidative damage in plaque VSMCs may be the result of decreased expression and acetylation of OGG1.

### OGG1 is a Major BER Enzyme in VSMCs

**Figure 2.**
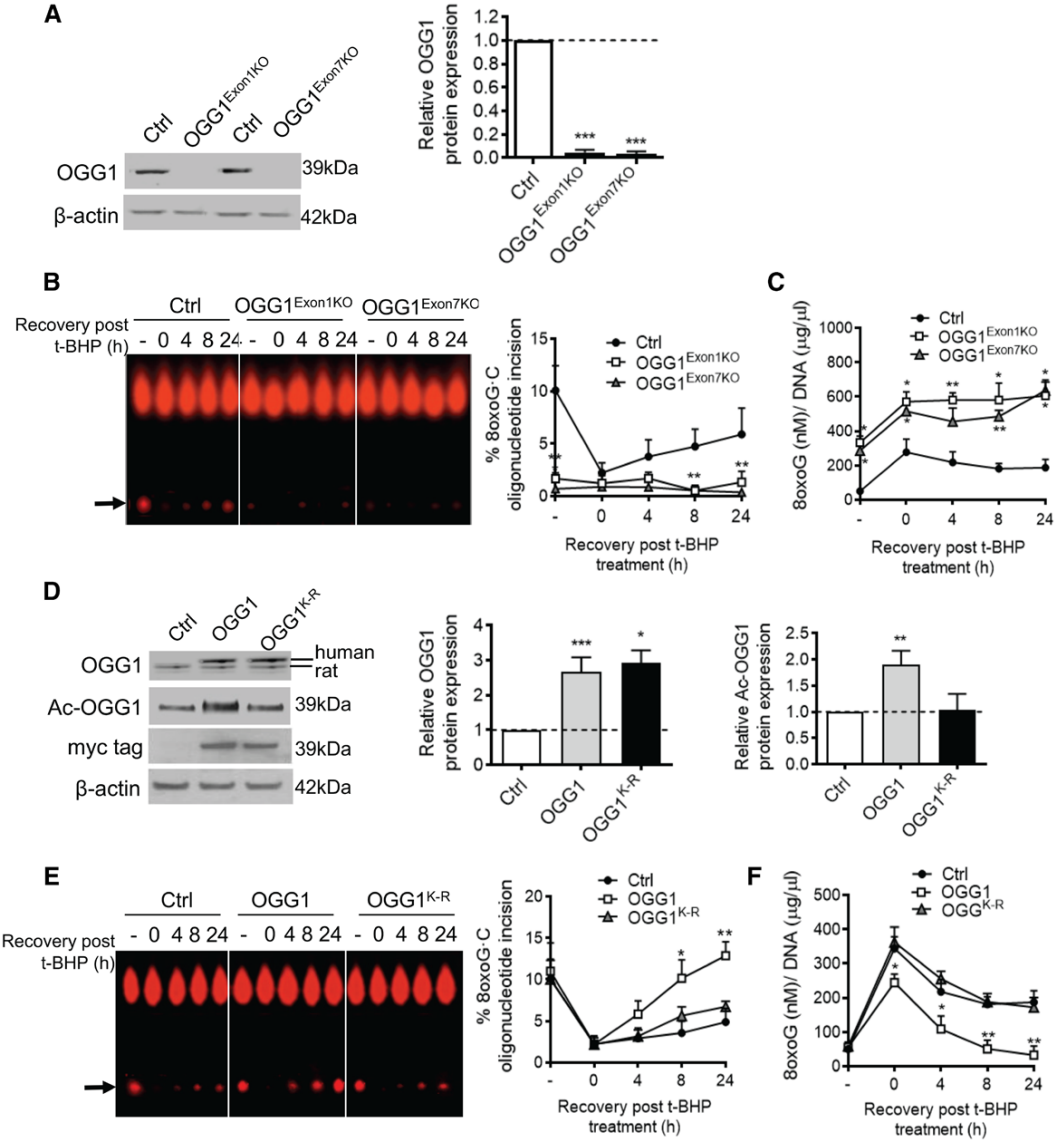
**OGG1 is the major BER enzyme for 8oxoG, and its activity is regulated by acetylation. A**, 8-Oxoguanine (8oxoG) DNA glycosylase I (OGG1) protein expression in control or OGG1 exon1 or exon7 CRISPR knockout rat vascular smooth muscle cell lines (n=4). **B**, Base excision repair (BER) assay analysis and (**C**) 8oxoG intracellular levels measured by ELISA in control (Ctrl), OGG1^Exon1KO^, or OGG1^Exon7KO^ rat vascular smooth muscle cells (VSMCs) at untreated baseline (-) or after tert-butyl hydroperoxide (t-BHP) treatment for 1 hour (t=0) and after t-BHP removal (4-, 8-, 24-hour recovery; n=5). **D**, Western blot of OGG1, acetylated OGG1 (Ac-OGG1), or myc tag in rat VSMCs expressing the empty vector (Ctrl), human OGG1 (OGG1), or acetylation mutant OGG1 (OGG1^K-R^). Immunoblotting for OGG1 shows a lower band for endogenous rat OGG1 and a higher band for exogenous human OGG1 (n=4). **E**, BER assay analysis and (**F**) 8oxoG intracellular levels measured by ELISA in Ctrl, OGG1, or OGG1^K-R^ cells at untreated baseline (-) or after t-BHP treatment for 1 hour (t=0) and after t-BHP removal (4-, 8-, 24-hour recovery; n=4). All graphical data are mean±SEM. *P<0.05, **P<0.01, ***P<0.001, 1-way ANOVA (Bonferroni post hoc).

To determine whether OGG1 is an important regulator of 8oxoG BER in VSMCs, we knocked down OGG1 in vitro using CRISPR/Cas9 to delete exon 1 or 7 in rat VSMCs. OGG1 expression was efficiently and stably reduced in OGG1^Exon1KO^ or OGG1^Exon7KO^ cells (Figure 2A), with no compensatory effect on expression of other BER enzymes such as NEIL1 and NTH (Figure IIa in the online-only Data Supplement). Oxidative DNA damage was stimulated by treatment with tert-butyl hydroperoxide (t-BHP) for 1 hour, which induces oxidative stress and 8oxoG in VSMCs.^15^ Cells were left to recover for 0 to 24 hours, and 8oxoG BER activity was examined. t-BHP transiently inhibited BER activity in control cells, which then normalized by 24 hours; OGG1 knockdown reduced both basal and t-BHP–induced 8oxoG BER activity, such that OGG1 was responsible for >95% of 8oxoG BER activity (Figure 2B). Consistent with t-BHP–induced inhibition of BER, t-BHP also increased intracellular 8oxoG content in VSMCs (Figure 2C), albeit with recovery incomplete by 24 hours. OGG1^Exon1KO^ and OGG1^Exon7KO^ cells showed higher basal and t-BHP–induced intracellular 8oxoG, which was completely unchanged at 24 hours (Figure 2C), confirming that OGG1 is a major BER enzyme repairing 8oxoG in VSMCs.

### Repair of 8oxoG Requires OGG1 Acetylation

To determine whether 8oxoG repair activity requires OGG1 acetylation in VSMCs, we stably expressed wild-type human OGG1, OGG1^K-^^R^, or the empty vector in rat VSMCs by retrovirus-mediated gene transfer. OGG1^K-R^ is an acetylation site mutant in which lysine 338 and 341 (which are active in 8oxoG repair^19^) are replaced by arginines. Exogenous human OGG1 was expressed at comparable levels in OGG1 and OGG1^K-R^ cells (Figure 2D), was localized to the nucleus (Figure IIb in the online-only Data Supplement), and did not suppress endogenous rat OGG1 expression (Figure 2D). However, Ac-OGG1 expression was increased in OGG1 but not OGG1^K-R^ VSMCs (Figure 2D). OGG1 but not OGG1^K-R^ cells showed significantly increased 8oxoG BER after t-BHP compared with control cells expressing the empty vector alone (Figure 2E). Control, OGG1, and OGG1^K-R^ VSMCs showed similar intracellular baseline 8oxoG and similar 8oxoG levels after 1 hour of t-BHP treatment; however, OGG1 but not OGG1^K-R^ VSMCs displayed more rapid removal of 8oxoG (Figure 2F). To exclude the possibility that expression of OGG1 altered ROS generation by t-BHP, we examined ROS levels at baseline and after t-BHP in the 3 cell lines; ROS levels were identical in control, OGG1, and OGG1^K-R^ VSMCs both at baseline and after t-BHP (Figure IIc in the online-only Data Supplement), indicating that OGG1 acetylation regulates efficiency of 8oxoG removal after oxidative stress without affecting ROS.

### OGG1 Acetylation and Stability Are Regulated by p300

**Figure 3.**
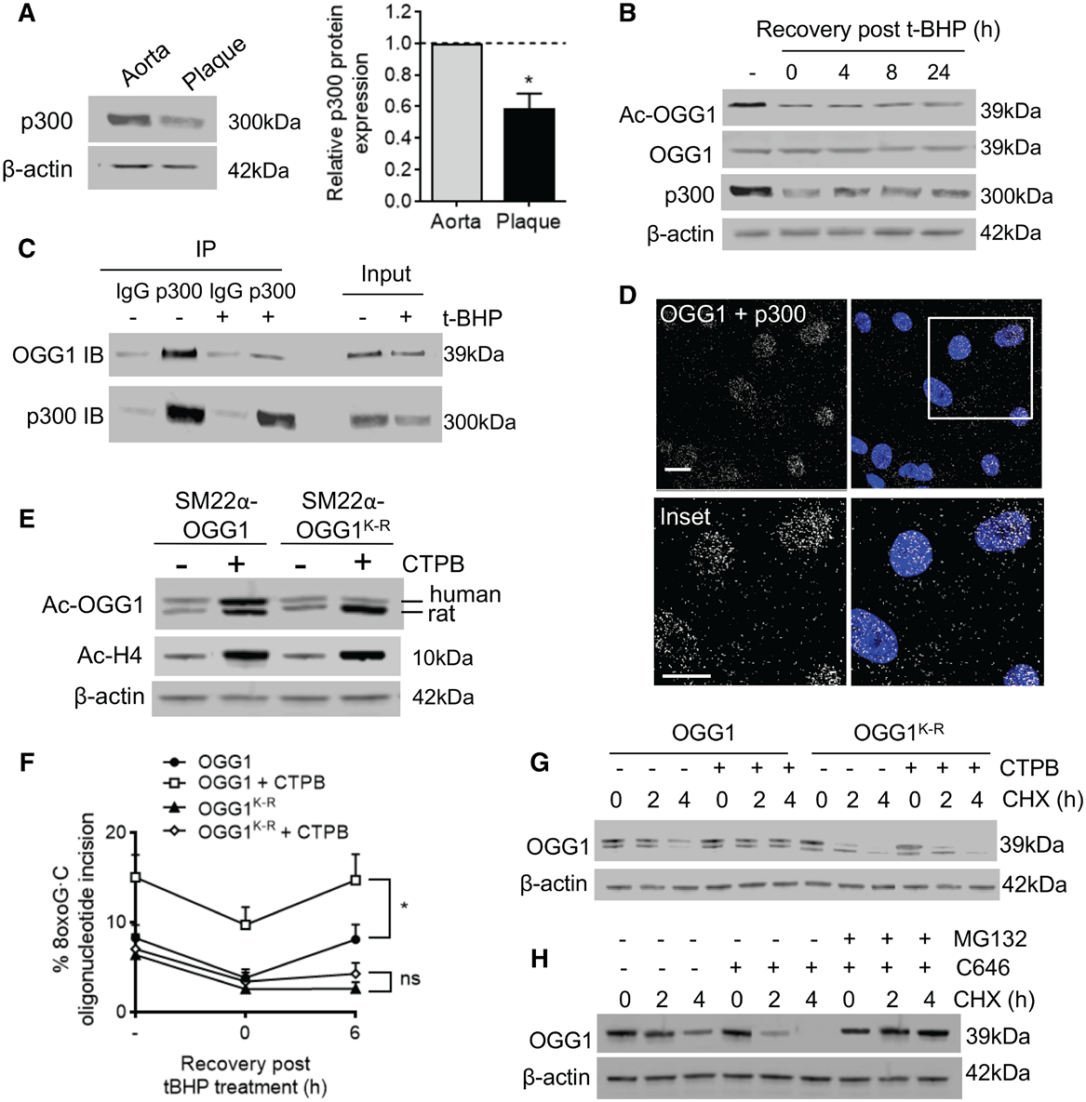
**p300 is downregulated in plaque VSMCs and regulates OGG1 acetylation and BER. A**, Western blot of p300 expression in cultured human plaque or normal aortic vascular smooth muscle cells (VSMCs; n=4). **B**, Western blot of 8-oxoguanine (8oxoG) DNA glycosylase I (OGG1), acetylated (Ac-) OGG1, and p300 levels in human VSMCs after 1 hour of tert-butyl hydroperoxide (t-BHP) treatment and 0- to 24-hour recovery (n=4). **C**, Immunoprecipitation (IP) of human vascular smooth muscle cell lysates with an anti-p300 antibody analyzed by immunoblotting (IB) with anti-OGG1 and anti-p300 antibodies (n=3). **D**, Proximity ligation assay of OGG1-p300 interaction with rabbit anti-OGG1 and mouse anti-p300 antibodies (n=3). Scale bar, 20µm. **E**, Western blot for Ac-OGG1 and Ac-histone 4 (H4) in VSMCs expressing OGG1 or OGG1^K-R^ with or without CTPB treatment (10 μmol/L; n=3). **F**, Quantification of base excision repair(BER) assay in OGG1 or OGG1^K-R^ VSMCs either untreated (-) or after 1 hour of t-BHP (0) or 6-hour recovery with/without CTPB (n=3). Representative gel is shown in Figure IVa in the online-only Data Supplement. **G**, Western blot of OGG1 expression after cycloheximide (CHX) treatment (0–4 hours) in OGG1 or OGG1^K-R^ VSMCs with/without the p300 activator CTPB (n=3). **H**, Western blot of OGG1 expression after CHX treatment (0–4 hours) in control cells with/without CTPB with/without the proteasomal degradation inhibitor MG132 (10 μmol/L; n=3). Scale bars, 25 µm. All graphical data are mean±SEM. *P<0.05, Student *t* test.

Although the acetyltransferase p300 can acetylate OGG1 in cancer cells,^19^ the major acetyltransferase and deacetylase enzymes of OGG1 in VSMCs are not known, nor is their expression in atherosclerosis. We examined p300 expression in cultured normal aortic and plaque VSMCs and whether VSMC OGG1 and 8oxoG repair activity was regulated by p300. Both p300 mRNA expression (Figure IIIa in the online-only Data Supplement) and protein expression (Figure 3A) were reduced in plaque compared with aortic VSMCs, and the percent of VSMCs expressing p300 was also reduced in plaque VSMCs in vivo (Figure IIIb in the online-only Data Supplement). To examine whether oxidative stress could be the underlying cause of reduced p300 levels in plaque VSMCs, we treated control VSMCs with t-BHP for 1 hour and examined OGG1, Ac-OGG1, and p300 protein expression up to 24 hours of recovery. t-BHP reduced both Ac-OGG1 and p300 expression in parallel, which did not normalize by 24 hours (Figure 3B and Figure IIIc in the online-only Data Supplement), suggesting that ROS-induced reduction of OGG1 acetylation is caused by the ROS-induced decrease in p300 expression. The reduced OGG1 acetylation and p300 expression even at 24 hours after t-BHP treatment (Figure 3B) may explain the incomplete 8oxoG repair seen in control cells (Figure 2C).

To examine whether p300 interacts with OGG1 at endogenous levels of expression in VSMCs, human VSMC lysates were immunoprecipated with an anti-p300 antibody and probed for OGG1. p300 associated with OGG1 at endogenous levels of expression, and p300 complex formation with OGG1 was reduced by t-BHP (Figure 3C). Proximity ligation assay confirmed the p300/OGG1 interaction in human VSMCs and localized it primarily to the nucleus (Figure 3D;negative controls shown in Figure IIId in the online-only Data Supplement). To determine whether p300 regulates OGG1 acetylation and BER, we examined Ac-OGG1 expression and BER activity in OGG1 or OGG1^K-R^ cells treated with 1 hour of t-BHP and 24-hour recovery with or without CTPB, a specific activator of p300 histone acetyltransferase activity.^20,21^ CTPB induced hyperacetylation of histone 4 as expected^22^ (Figure 3E). CTPB induced acetylation of endogenous rat and exogenous human OGG1 in OGG1 cells, but human OGG1 remained unchanged in OGG1^K-R^ cells (Figure 3E). CTPB also increased 8oxoG repair activity in OGG1 cells but not in OGG1^K-R^ cells (Figure 3F and Figure IVa in the online-only Data Supplement), in line with the effects on OGG1 acetylation. Furthermore, the p300 inhibitor C646^23^ inhibited BER in VSMCs (Figure IVb in the online-only Data Supplement).

Our finding that OGG1 mRNA is increased in human plaque VSMCs but expression of OGG1 and acetylated OGG1 protein are reduced suggests that both expression and activity of OGG1 are controlled by posttranslational mechanisms. We therefore examined the stability of OGG1 protein in cells treated with cycloheximide to prevent de novo protein synthesis. Cycloheximide reduced expression of endogenous rat OGG1 in control, OGG1, and OGG1^K-R^ VSMCs to a similar extent; in contrast, the reduction in human OGG1 was slower in OGG1 cells compared with OGG1^K-R^ VSMCs, suggesting that acetylation of OGG1 promotes its stability (Figure IVc in the online-only Data Supplement). To determine whether p300 regulates OGG1 stability, we repeated this experiment with and without the p300 activator CTPB. CTPB enhanced the stability of OGG1 in OGG1 cells but not in OGG1^K-R^ cells (Figure 3G and Figure IVd in the online-only Data Supplement). We also tested whether p300 regulates OGG1 stability through its degradation. OGG1 stability was reduced in the presence of the p300 inhibitor C646, but OGG1 expression was restored after additional administration of the proteasomal inhibitor MG132 (Figure 3H and Figure IVe in the online-only Data Supplement), indicating that p300 regulates OGG1 expression and proteasome-mediated degradation through its acetylation.

### SIRT1 is an OGG1 Deacetylase in VSMCs

The deacetylase responsible for inhibiting OGG1 activity in VSMCs is unknown because, although OGG1 interacts with class I histone deacetylases, previous studies showed that the activity was not affected by the SIRT1 inhibitor nicotinamide.^19^ We therefore examined whether VSMC OGG1 activity and BER activity were regulated by SIRT1.

**Figure 4.**
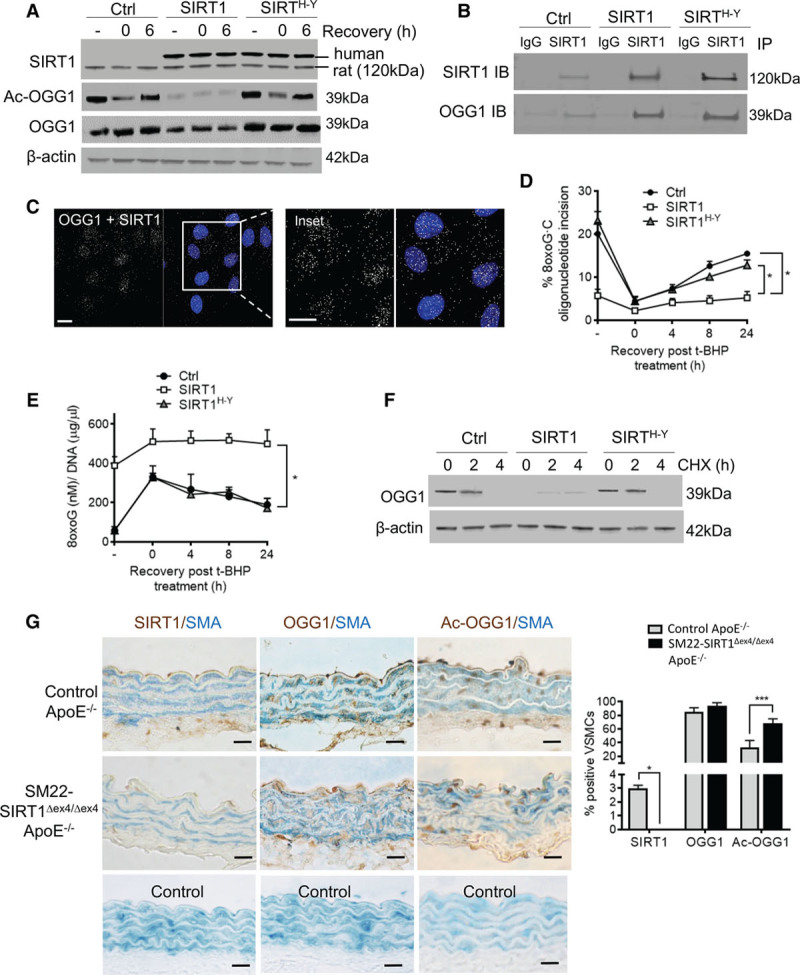
**SIRT1 binds OGG1 and regulates deacetylation of OGG1 in vitro and in vivo. A**, Western blot of total sirtuin 1 (SIRT1), acetylated (Ac-) 8-oxoguanine (8oxoG) DNA glycosylase I (OGG1), and OGG1 expression in rat vascular smooth muscle cells (VSMCs) expressing the empty vector (control [Ctrl]), human SIRT1 (SIRT1), or deacetylase defective mutant SIRT^H364Y^ cells after 1 hour of tert-butyl hydroperoxide (t-BHP) treatment and after 0- to 6-hour recovery (n=3). Immunoblotting for SIRT1 shows a lower band for endogenous rat SIRT1 and a higher band for exogenous human SIRT1. **B**, Immunoprecipitation (IP) of human vascular smooth muscle cell lysates with an anti-SIRT1 antibody analyzed by immunoblotting (IB) with anti-SIRT1 and anti-OGG1 antibodies (n=3). **C**, Proximity ligation assay of OGG1-SIRT1 interaction with rabbit anti-OGG1 and mouse anti-SIRT1 antibodies (n=3). **D**, Quantification of base excision repairassay in Ctrl, SIRT1, or SIRT^1H-Y^ VSMCs either untreated (-) or after 1 hour of t-BHP (0) or 0- to 24-hour recovery (n=3). Representative gel is shown in Figure Vb in the online-only Data Supplement. **E**, 8oxoG intracellular levels measured by ELISA in control, SIRT1, or SIRT^1H-Y^ VSMCs after 1 hour of t-BHP treatment and after 0- to 24-hour recovery (n=3). **F**, Western blot of OGG1 expression after cycloheximide (CHX; 0–4 hours) treatment of control, SIRT1, or SIRT^1H-Y^ VSMCs (n=3). **G**, Immunohistochemistry for SIRT1, OGG1, or Ac-OGG1 (brown) in aortas from control ApoE^−/−^ or SIRT1^−/−^/ApoE^−/−^ mice (n=10). Tissue sections were also costained for α-smooth muscle actin (SMA; blue). Negative control sections with isotype-matched antibodies are shown below. Scale bar, 25 µm. All graphical data are mean±SEM. *P<0.05, ***P<0.001, 1-way ANOVA (Bonferroni post hoc).

To determine whether OGG1 interacts with and is deacetylated by SIRT1, we generated VSMCs that stably express wild-type human SIRT1 or the deacetylase-deficient mutant SIRT1^H365Y^ by retrovirus-mediated gene transfer.^10^ VSMCs expressing the empty vector (control), SIRT1, or SIRT1^H-Y^ were treated with t-BHP and SIRT1, and Ac-OGG1 and OGG1 protein expression was examined. SIRT1 but not SIRT1^H-Y^ reduced Ac-OGG1 and total OGG1 expression (Figure 4A), suggesting that OGG1 is a SIRT1 deacetylation substrate. Indeed, SIRT1 coimmunoprecipitated with OGG1 both at endogenous levels and when overexpressed (Figure 4B), and proximity ligation assay in human VSMCs confirmed this protein interaction at endogenous levels (Figure 4C and Figure Va in the online-only Data Supplement). Consistent with SIRT1 reducing OGG1 acetylation, SIRT1 but not SIRT1^H-Y^ reduced 8oxoG repair activity (Figure 4D and Figure Vb in the online-only Data Supplement) and increased intracellular 8oxoG content (Figure 4E). SIRT1 but not SIRT1^H-Y^ also reduced OGG1 stability (Figure 4F), consistent with SIRT1 deacetylation of OGG1. Because SIRT1 is both regulated by redox-mediated mechanisms and can inhibit ROS generation (reviewed previously^24,25^), the differences in 8oxoG levels after t-BHP could be caused by different levels of ROS induced in each cell type; however, ROS levels in control, SIRT1, or SIRT1^H-Y^ cells after t-BHP were similar (Figure Vc in the online-only Data Supplement). Finally, we examined the expression of OGG1 and Ac-OGG1 in aortas from ApoE^−/−^ mice that express a truncated inactive SIRT1 (SIRT1^Δex4/Δex4^) from the SMC-specific SM22α (transgelin [*Tagln*]) promoter.^10^ SIRT1 expression was detectable in <1% of VSMCs in SM22-SIRT1^Δex4/Δex4^ ApoE^−/−^ mice, confirming the efficacy of the recombination. OGG1 expression was seen at similar levels in SM22-SIRT1^Δex4/Δex4^ ApoE^−/−^ and control mice; in contrast, Ac-OGG1 expression was increased in SM22-SIRT1^Δex4/Δex4^ ApoE^−/−^ mice, consistent with SIRT1 deacetylation of OGG1 (Figure 4G). Together these data indicate that SIRT1 binds to and regulates OGG1deacetylation and stability and thus regulates oxidative damage in VSMCs.

### VSMC OGG1 Reduces Oxidative Damage and Atherosclerosis In Vivo

Whole-body or bone marrow cell–restricted knockout of OGG1 results in increased atherosclerosis in low-density lipoprotein receptor–null mice.^8^ However, OGG1 knockout results in massive 8oxoG accumulation, and the effects of endogenous levels of 8oxoG found in atherosclerosis and VSMC OGG1 are not known. We therefore generated mice expressing myc-tagged human OGG1 or OGG1^K-R^ from the minimal SM22α promoter (SM22α-OGG1 or SM22α-OGG1^K-R^ mice). This promoter is expressed in VSMCs in large arteries only^26^ and has an additional deletion of the CARG motif to prevent promoter downregulation during VSMC phenotypic change in atherosclerosis.^27^ SM22α-OGG1 and SM22α-OGG1^K-R^ mice were compared against control mice and OGG1^−/−^ mice.^9^ Mouse OGG1 mRNA expression was markedly reduced in all tissues in OGG1^−/−^ mice but was not affected by coexpression of human OGG1 in SM22α-OGG1 or SM22α-OGG1^K-R^ mice (Figure VIa in the online-only Data Supplement). Previous studies have also shown that the full-length SM22α promoter is also expressed in myeloid cells^28^; in contrast, the minimal SM22α promoter–driven human OGG1 transgene was expressed only in aortas of SM22α-OGG1 mice but not peripheral blood, bone marrow cells, or spleen (Figure VIb in the online-only Data Supplement).

**Figure 5.**
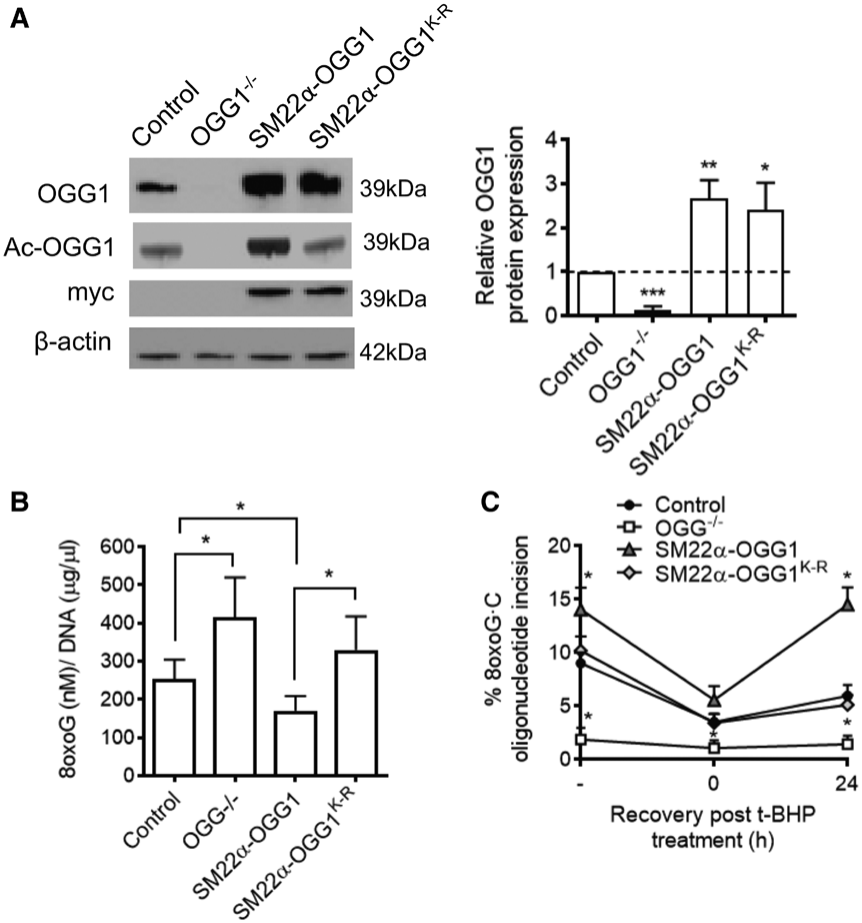
**OGG1 regulates 8oxoG expression in VSMCs in vivo and BER. A**, Western blot of 8-oxoguanine (8oxoG) DNA glycosylase I (OGG1), acetylated (Ac-) OGG1, and myc tag in vascular smooth muscle cells (VSMCs) cultured from wild-type (control), OGG1^−/−^, SM22α-OGG1, or SM22α-OGG1^K-R^ mouse aortas (n=4). **B**, 8oxoG intracellular levels measured by ELISA in control, OGG1^−/−^, SM22α-OGG1, or SM22α-OGG1^K-R^ VSMCs (n=4). **C**, Base excision repair(BER) assay quantification in control, OGG1^−/−^, SM22α-OGG1, or SM22α-OGG1^K-R^ VSMCs treated with tert-butyl hydroperoxide (t-BHP) for 1 hour and recovered for up to 24 hours (n=4). All graphical data are mean±SEM. *P<0.05, 1-way ANOVA (Bonferroni post hoc).

OGG1 protein expression was markedly reduced in VSMCs cultured from OGG1^−/−^ mice but increased in SM22α-OGG1 and SM22α-OGG1^K-R^ mice to similar levels (Figure 5A). Ac-OGG1 expression was increased in SM22α-OGG1 mouse VSMCs but not SM22α-OGG1^K-R^ VSMCs, whereas myc tag expression was observed only in transgenic lines (Figure 5A). ELISA of cultured VSMCs from these mice showed increased 8oxoG in OGG1^−/−^ mice and reduced 8oxoG in SM22α-OGG1 but not SM22α-OGG1^K-R^ mice (Figure 5B). 8oxoG repair activity was increased in SM22α-OGG1 VSMCs, reduced in OGG1^−/−^ VSMCs, but unchanged in SM22α-OGG1^K-R^ VSMCs compared with control cells (Figure 5C and Figure VIc in the online-only Data Supplement). Thus, OGG1^−/−^ mice allow examination of the effect of supraphysiological levels of 8oxoG in all tissues on atherosclerosis; SM22α-OGG1 mice allow determination of the effect of rescuing OGG1 activity and suppressing endogenous levels of 8oxoG in VSMCs only; and SM22α-OGG1^K-R^ mice provide the acetylation-mutant control for SM22α-OGG1 mice.

**Figure 6.**
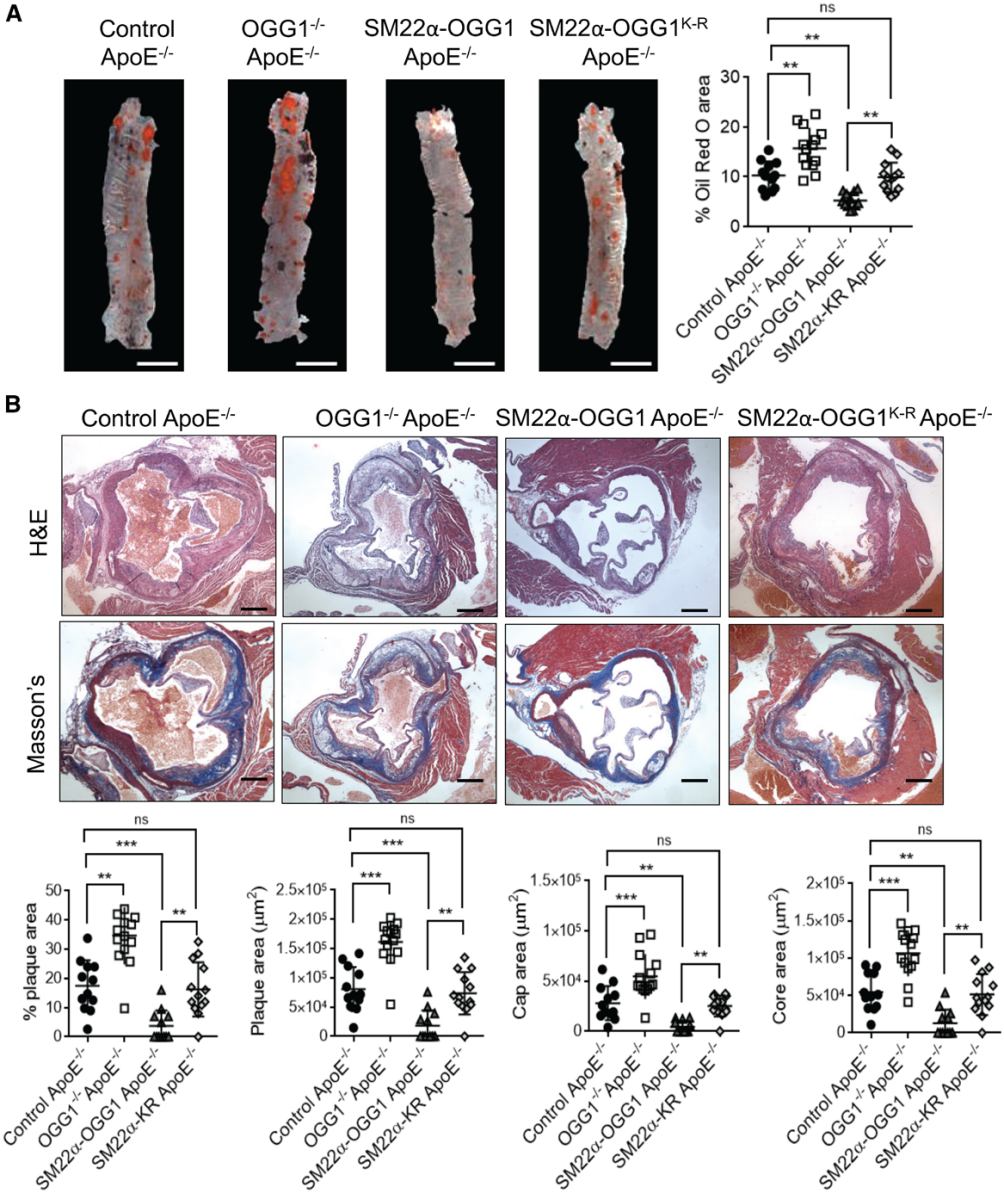
**Effects of 8-oxoguanine DNA glycosylase I (OGG1) on plaque development and morphology in vivo. A**, Representative images and quantification of en face preparations of descending aortas from control ApoE^−/−^ (n=12), OGG1^−/−^ ApoE^−/−^ (n=14), SM22α-OGG1 ApoE^−/−^ (n=12), and SM22α-OGG1^K-R^ ApoE^−/−^ (n=13) mice stained with Oil Red O. Scale bar, 2 mm. **B**, Hematoxylin-eosin (H&E) and Masson trichrome immunohistochemistry of control ApoE^−/−^ (n=12), OGG1^−/−^ ApoE^−/−^ (n=14), SM22α-OGG1 ApoE^−/−^ (n=12), and SM22α-OGG1^K-R^ ApoE^−/−^ (n=13) mouse aortic roots at 22 weeks after fat feeding from 8 to 22 weeks. Quantification of percent plaque size, plaque area, core area, and cap area (micrometers squared). Scale bar, 200 µm. All graphical data are mean±SEM. **P<0.01, ***P<0.001, 1-way ANOVA (Bonferroni post hoc).

Control wild-type, OGG1^−/−^, SM22α-OGG1, and SM22α-OGG1^K-R^ mice were crossed onto an ApoE^−/−^ background, weaned at 6 weeks, and male and female littermates were fat fed from 8 to 22 weeks of age. Weight gain (Figure VIIa in the online-only Data Supplement); serum lipids (Figure VIIb in the online-only Data Supplement); systolic, diastolic, and mean arterial pressures; and heart rates were similar across all groups before and after fat feeding (Figure VIIc in the online-only Data Supplement). Atherosclerosis was increased in the descending aorta and aortic root in OGG1^−/−^ ApoE^−/−^ mice compared with controls (Figure 6A and 6B) but markedly decreased in both vascular beds in SM22α-OGG1 ApoE^−/−^ mice; this protective effect was lost in SM22α-OGG1^K-R^ ApoE^−/−^ mice. Both necrotic core and fibrous cap areas were reduced in SM22α-OGG1 ApoE^−/−^ mice (Figure 6C) compared with controls, with no overall change in relative proportions of the necrotic core and fibrous cap to the plaque or to each other (Figure VIIIa in the online-only Data Supplement). Analyses of male and female mice were grouped together (Figure VIIIb in the online-only Data Supplement) because we found no significant differences between sexes for any parameters measured except for body weight (not shown). Aortic plaques in SM22α-OGG1 mice had reduced 8oxoG levels compared with SM22α-OGG1^K-R^ and control mice, correlating with reduced plaque area, and significantly fewer terminal UTP nick end-labeling–positive apoptotic cells compared with SM22α-OGG1^K-R^ mice (Figure VIIIc in the online-only Data Supplement).

### Oxidative DNA Damage Accumulates at Telomeres; Induces Strand Breaks, Cell Senescence, and Apoptosis; and Drives Inflammation

**Figure 7.**
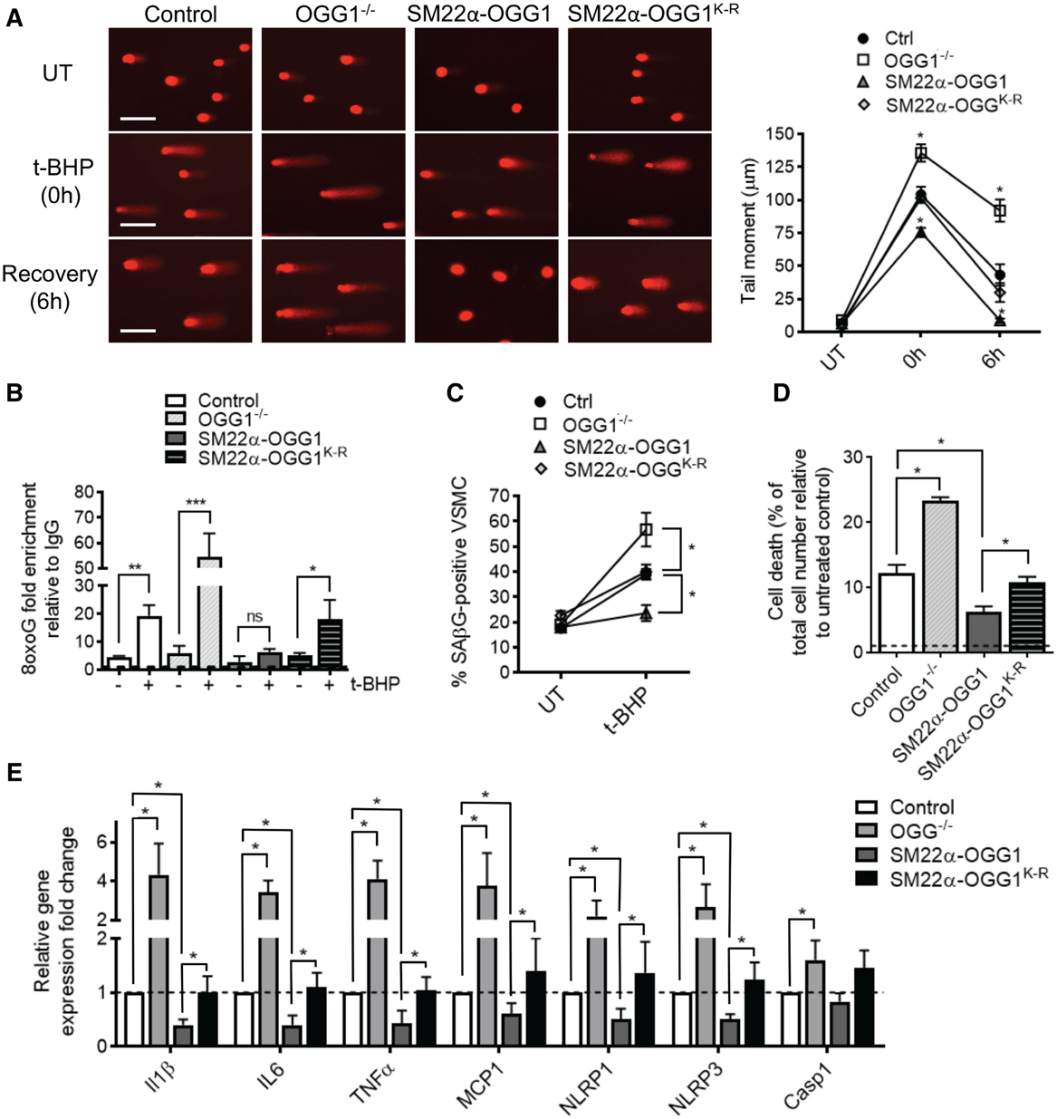
**8-Oxoguanine (8oxoG) DNA glycosylase I (OGG1) regulates DNA strand breaks, cell senescence, apoptosis, and inflammasome pathways. A**, Comet assay with quantification for cultured vascular smooth muscle cells (VSMCs) from wild-type control, OGG1^−/−^, SM22α-OGG1, or SM22α-OGG1^K-R^ mice treated with tert-butyl hydroperoxide (t-BHP) for 1 hour and recovered for 6 hours (n=3). Scale bar, 100 µm. UT indicates untreated. **B**, Chromatin immunoprecipitation–quantitative polymerase chain reaction using primers to a telomeric region on 8oxoG-bound chromatin from control, OGG1^−/−^, SM22α-OGG1, or SM22α-OGG1^K-R^ mouse VSMCs (n=3). Data are shown as fold-change over immunoglobulin G (IgG). **C**, Percent VSMCs expressing senescence-associated β-galactosidase activity (SaβG) from wild-type control, OGG1^−/−^, SM22α-OGG1, or SM22α-OGG1^K-R^ mice (n=3). **D**, Apoptosis assayed by annexin V and propidium iodide staining of mouse cells with/without t-BHP treatment by flow cytometry (n=3). **E**, Relative expression of transcripts for inflammatory cytokines and inflammasome-associated components in aortic arch tissue from control ApoE^−/−^, OGG1^−/−^ ApoE^−/−^, SM22α-OGG1 ApoE^−/−^, and SM22α-OGG1^K-R^ ApoE^−/−^ mice after fat feeding (n=4). All graphical data are mean±SEM. *P<0.05, **P<0.01, ***P<0.001, 1-way ANOVA (Bonferroni post hoc).

Although VSMC OGG1 protected against atherosclerosis, the underlying mechanisms, which may be multiple, are unclear. For example, defects in BER can cause DNA strand breaks, resulting in cell death. Indeed, OGG1^−/−^ VSMCs showed increased DNA strand breaks after t-BHP treatment with a reduced rate of repair; DNA damage was lower in SM22α-OGG1 but not SM22α-OGG1^K-R^ VSMCs versus control cells (Figure 7A). 8oxoG accumulation at telomeres can result in cell senescence and subsequent inflammation. Indeed, chromatin immunoprecipitation–quantitative polymerase chain reaction showed that OGG1^−/−^ VSMCs had increased 8oxoG accumulation at telomeres, and this was reduced in SM22α-OGG1 but not SM22α-OGG1^K-R^ cells (Figure 7B). The percent of cells expressing senescence-associated β-galactosidase after t-BHP was increased in OGG1^−/−^ VSMCs and reduced in SM22α-OGG1 but not SM22α-OGG1^K-R^ cells (Figure 7C and Figure IX in the online-only Data Supplement). Finally, t-BHP–induced cell death was increased in OGG1^−/−^ and reduced in SM22α-OGG1 but not SM22α-OGG1^K-R^ cells (Figure 7D), indicating that OGG1 overexpression protects VSMCs against apoptosis, but this depends on OGG1 acetylation.

Oxidized DNA can also activate the inflammasome, resulting in release of proinflammatory cytokines such as interleukin (IL)-1β.^8^ Using quantitative polymerase chain reaction, we profiled aortic arch tissue from ApoE^−/−^ mice after high-fat diet feeding for levels of inflammatory cytokines and inflammasome components. SM22α-OGG1 ApoE^−/−^ aortic tissue contained significantly decreased levels of transcripts for cytokines IL-1β, IL-6, C-C motif chemokine ligand/monocyte chemotactic protein 1, and tumor necrosis factor-α and inflammasome components NLRP1 and NLRP3 (Figure 7E), indicative of a shift toward a more anti-inflammatory phenotype, with an opposite profile seen in OGG1^−/−^ ApoE^−/−^ mice. Serum levels of IL-1β, IL-6, C-C motif chemokine ligand/monocyte chemotactic protein 1, and tumor necrosis factor-α were also decreased in SM22α-OGG1 ApoE^−/−^ mice after high-fat diet feeding compared with controls (Figure Xa in the online-only Data Supplement). To identify the source of these cytokines, we cultured VSMCs from these mice and determined cytokine secretion after t-BHP treatment for 24 hours. t-BHP did not increase levels of IL-1β, IL-6, monocyte chemotactic protein 1,and tumor necrosis factor-α in conditioned media of SM22α-OGG1 ApoE^−/−^ cells compared with SM22α-OGG1^K-R^ ApoE^−/−^ and control ApoE^−/−^ cells (Figure Xb in the online-only Data Supplement). The anti-inflammatory profile in vivo of OGG1 mice and in culture of OGG1 VSMCs suggests that 8oxoG in VSMCs may exert a direct proinflammatory effect on the vessel wall.

## Discussion

Advanced human atherosclerotic plaques demonstrate extensive 8oxoG accumulation in multiple cell types,^5,6^ and polymorphisms in some BER enzymes are associated with myocardial infarction,^29^ suggesting that impaired BER might promote plaque development or instability. Indeed, OGG1 knockout in hematopoietic cells promotes atherogenesis and enhanced inflammasome activation in macrophages.^8^ However, VSMCs are long-lived cells,^30^ and the effects of oxidative DNA damage in VSMCs in atherosclerosis are not known. In particular, chronic oxidative stress can increase 8oxoG levels >250-fold without apparent severe consequences,^31^ and OGG1^−/−^ mice are born and develop normally with a normal life span, despite a 7-fold increase in 8oxoG in nuclear DNA and a >20-fold increase in mitochondrial DNA.^9,32–34^ More controversially, some studies suggest that 8oxoG may actually protect against inflammation-induced DNA damage.^35–37^

Our study reports a number of novel findings. In particular, we demonstrate the following: evidence of defective 8oxoG BER in human plaque VSMCs, not just increased damage or reduced expression of BER enzymes; that reducing physiological 8oxoG levels reduces atherosclerosis, in addition to the detrimental effects of artificially elevated 8oxoG; the importance of OGG1 acetylation to its function in vivo; that SIRT1 regulates OGG1 acetylation in vivo; and that p300 regulates OGG1 stability, not just activity. Specifically, we find that OGG1 is a major BER enzyme in VSMCs, the activity and protein stability of which are regulated by acetylation. Nuclear 8oxoG repair is defective in human atherosclerotic plaque VSMCs, associated with reduced acetylation of OGG1, which results in proteasome-mediated OGG1 degradation and reduced activity. Our findings identify that atherosclerosis is a disease characterized by defective BER in VSMCs and that endogenous levels of oxidative DNA damage in VSMCs promote plaque development.

Human atherosclerotic plaques show a variety of DNA damage, including single- and double-strand breaks, telomere shortening, oxidative DNA damage, and mutations (reviewed by Uryga et al^7^). Plaque VSMCs also show differences in expression of multiple DNA damage repair proteins, indicative of activation of a DNA damage repair response.^11^ Some human DNA damage syndromes are associated with premature atherosclerosis, and previous studies in mice have shown that knockout of some DNA repair enzymes can promote atherosclerosis or vascular dysfunction,^8,38^ suggesting that DNA damage may promote atherosclerosis. Indeed, a recent study showed downregulation of OGG1 transcripts in human plaque tissue compared with normal vessels.^8^ In contrast, we found that OGG1 mRNA is increased in human plaque VSMCs, whereas total OGG1 protein expression is decreased compared with aortic VSMCs. This difference is likely the result of the analyses being performed in whole-plaque tissue,^8^ which contains a heterogeneous mixture of cells, versus VSMC cultures in this study. However, this is the first study to demonstrate that human atherosclerosis exhibits a DNA repair defect in VSMCs; that the defect is caused by reduced expression, acetylation, and activity of a specific DNA repair protein; and that correction of that defect in VSMCs alone can markedly reduce plaque formation.

VSMCs in the normal vessel wall are characterized by low rates of cell division, cell death, and cell senescence and maintain a contractile phenotype that expresses low levels of inflammatory cytokines (reviewed by Garrido and Bennett^39^). In contrast, VSMCs in atherosclerotic plaques or cultured from plaques show higher rates of cell death and cell senescence, accompanied by secretion of a variety of proinflammatory cytokines, some of which may be the result of cell senescence.^40,41^ Cell death and cell senescence promote both plaque development and plaque progression in atherosclerosis.^42,43^ Oxidative DNA damage may affect multiple processes within VSMCs, and we show that VSMCs lacking OGG1 show increased cell death, cell senescence, and expression of inflammasome components, all of which are proatherogenic and all of which can be inhibited by overexpression of OGG1 after oxidative stress. Loss of OGG1 also increases oxidized mitochondrial DNA, inflammasome activation, and apoptosis in macrophages.^8^ However, the profound reduction in plaque formation in SM22α-OGG1 compared with control mice demonstrates that endogenous levels of 8oxoG found in VSMCs in atherosclerosis promote plaque development and emphasizes the importance of oxidative DNA damage–mediated activation of cell death and senescence in VSMCs and of VSMC-driven inflammation in atherogenesis.

Our data also demonstrate the complex effects of oxidative stress in DNA repair. Acetylation regulates multiple protein properties such as stability, localization, function, and protein-protein interactions.^44^ OGG1-mediated BER activity is regulated by acetylation of lysines K338/K341 (shown by Bhakat et al^19^ and here). Previous work in mouse embryonic fibroblasts showed decreased OGG1^K-R^ repair activity in vitro^19^ but not the total loss of activity we observe in VSMCs, implying that acetylation may be a more important modification for OGG1 activity in VSMCs compared with other cell types. We also show that p300 and SIRT1 are major acetyltransferase and deacetylase enzymes, respectively, for OGG1 acetylation, but the mechanisms of their effects and the effects of oxidative stress may differ between cells. For example, although p300 can interact with OGG1 in some cell types,^19^ oxidative stress enhances histone acetyltransferase activity of p300 in lung cells with no change in expression; in contrast, p300 protein expression was downregulated after oxidative stress in VSMCs. Consistent with previous studies,^19^ ROS also transiently decreased OGG1 activity directly, which may be caused by the reduced state of the redox-sensitive residues that determine its glycosylase activity.^45,46^ Our findings emphasize the detrimental effect of chronic oxidative stress in atherosclerosis through a positive feedback loop to generate further oxidative DNA damage. ROS induce oxidative DNA damage but also impair 8oxoG BER by downregulating OGG1 directly and indirectly through inhibiting p300 expression and thus OGG1 acetylation and protein stability. This finding may explain the observation that oxidative DNA damage persists much longer in atherosclerosis than other forms of DNA damage, even when the proatherogenic stimulus is removed.^5^

Although the regulation of OGG1 activity by acetylation is known,^19^ the identity of the OGG1 deacetylase has not been proven. SIRT1 is a NAD^+^-dependent lysine deacetylase with roles including cell aging, genomic stability, and cell apoptosis,^47^ but it has been excluded previously because the SIRT1 inhibitor nicotinamide did not increase OGG1 acetylation in HCT116 cells.^19^ However, an association of SIRT1 and OGG1 was not tested directly, and we have found that SIRT1 substrates are tissue specific.^10^ We identify that SIRT1 is a major enzyme regulating OGG1 activity, and its activity depends on its ability to deacetylate OGG1. Thus, SIRT1 but not SIRT1^HY^ reduces OGG1 acetylation, reduces 8oxoG repair, and promotes persistence of oxidative DNA damage. SIRT1 deletion in vivo also increases Ac-OGG1, indicating that SIRT1 can regulate OGG1 activity at endogenous levels. However, SIRT1 is downregulated in human atherosclerotic plaques and in plaque and senescent VSMCs compared with normal VSMCs in culture,^10^ suggesting that SIRT1 regulation of OGG1 may be particularly relevant in normal arteries as a way of deactivating OGG1 when oxidative DNA repair is complete, but it may be less important in atherosclerosis in which the reduced p300 expression results in reduced acetylated OGG1. However, SIRT1 also activates other components of the BER pathway, including the enzymes thymine DNA glycosylase, apurinic/apyrimidinic endonuclease 1, and poly(ADP-ribose) polymerase 1,^48,49^ which may partly explain the profound effect of VSMC SIRT1 knockout in atherosclerosis.^10^

**Figure 8.**
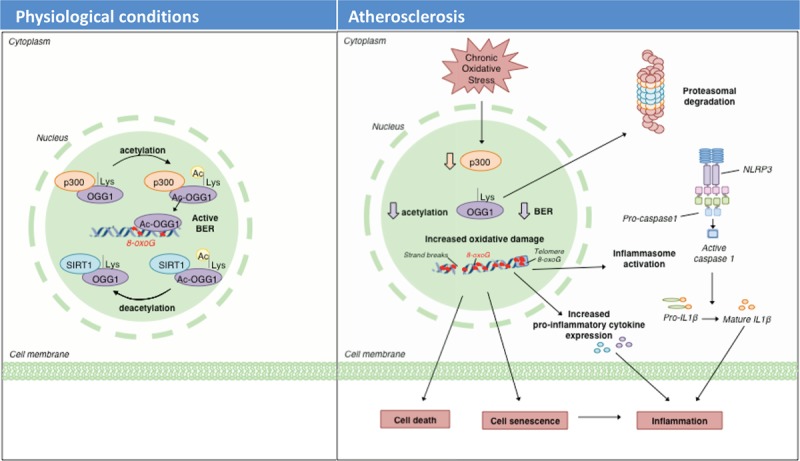
**Model of 8-oxoguanine (8oxoG) DNA glycosylase I (OGG1) regulation in VSMCs in atherosclerosis.** Ac indicated acetylated; BER, base excision repair; IL, interleukin; Lys, lysine; and SIRT1, sirtuin 1.

Our findings are consistent with the following model (Figure 8). OGG1 is preferentially acetylated by p300 in acute oxidative stress conditions that require 8oxoG repair activity, leading to efficient 8oxoG BER. Ac-OGG1 is deacetylated by SIRT1 when 8oxoG BER is complete. However, chronic oxidative stress in atherosclerosis results in downregulation of p300 and p300-mediated acetylation of OGG1, which in turn reduces OGG1 protein stability and thus expression and overall 8oxoG repair activity. ROS may also reduce OGG1 activity directly via effects on redox-sensitive residues and glycosylase activity.^45,46^ 8oxoG accumulation in VSMCs results in DNA strand breaks, cell senescence, cell death, inflammasome activation, and increased secretion of proinflammatory cytokines, all of which promote atherogenesis. Rescue of VSMC OGG1 prevents 8oxoG accumulation and these consequences, protecting against atherosclerosis.

## Conclusions

We have identified that 8oxoG repair is reduced in VSMCs in human atherosclerosis as a result of chronic oxidative stress–induced reduction in expression, stability, acetylation, and activity of OGG1. SIRT1 is also a major regulator of OGG1 acetylation, expression, and activity. OGG1 protects VSMCs against oxidative DNA damage, cell senescence, and apoptosis and reduces atherosclerosis formation, identifying BER as a possible therapeutic target in atherosclerosis.

## Sources of Funding

This work was funded by grants from the British Heart Foundation (RG/13/14/30314 and PG/11/112/29272), the Oxbridge British Heart Foundation Center for Regenerative Medicine (RM/13/3/30159), the British Heart Foundation Center for Cardiovascular Research Excellence, and the National Institute for Health Research Cambridge Biomedical Research Center.

## Disclosures

None.

## Supplementary Material
