# Supplementary material for: Defective Base Excision Repair of Oxidative DNA Damage in Vascular Smooth Muscle Cells Promotes Atherosclerosis
Source: Circulation. 2018 Oct 1;138(14):1446–62. doi: 10.1161/CIRCULATIONAHA.117.033249 (PMC6053042; doi:10.1161/CIRCULATIONAHA.117.033249)
Supplement: Supplementary file 1 [file cir-138-1446-s001.pdf]

SUPPLEMENTAL MATERIAL

SUPPLEMENTAL METHODS

Generation of transgenic mice
To produce transgenic mouse targeting constructs, myc-tagged hOGG1 or $OGG1^{K\text{-}R}$ cDNA was subcloned between the minimal SM22α promoter and a polyA sequence in pBluescript. The SM22α-OGG1 and SM22α-$OGG1^{K\text{-}R}$ transgenes were digested with BssHII and the transgene-containing fragment purified from an agarose gel. Transgenic mice were generated by pro-nuclear injection of SM22α-OGG1 and SM22α-$OGG1^{K\text{-}R}$ fragments into C57Bl6 embryos, and positive progeny used to generate individual lines.

Genotyping
Ear notches from transgenic mice were incubated with Chelex/Proteinase K for 2h at 56˚C. Following heat inactivation at 95˚C, 2 µl DNA was used in the subsequent PCR reaction. $ApoE^{-/-}$ genotyping was conducted according to Jackson Labs protocols and cycling conditions: 94˚C for 10 min, followed by 40 cycles of 94˚C for 45s, 63˚C for 45s and 72˚C for 90s. OGG1 genotyping was performed using the following cycling conditions: 95˚C for 5 min, followed by 40 cycles of 95˚C for 1 min, 62˚C for 2 min and 72˚C for 90s. SM22α-OGG1 transgene expression was analysed using primers that amplify a 550 bp region of OGG1 fused to the C-terminal myc tag using the following cycling conditions: 95˚C for 5 min, followed by 40 cycles of 94˚C for 1 min, 57˚C for 1 min and 72˚C for 1 min. PCR products were analysed on a 2% agarose gel containing ethidium bromide. Oligonucleotide sequences used for genotyping are listed in **Supplementary Table S1**.

For heterozygosity genotyping, digested ear notches were diluted 1:10 and underwent standard 2 step qPCR (Rotor-gene Q). Briefly, 3µl DNA was used in a qPCR reaction containing 2x PCR mastermix (Life Technologies), 20x human OGG1 or mouse GAPDH Taqman primer-probes (Life Technologies) and H_2O. Samples were analysed for OGG1 gene expression and normalized to GAPDH expression. Heterozygosity was defined by analysing the OGG1:GAPDH ratio whereby no expression corresponded to null animals; heterozygous animals had gene expression at 50% of the value of homozygous-positive animals.

Blood pressure analysis and cytokine detection
Blood pressure was determined by tail cuff measurements. V-PLEX Mouse Proinflammatory Panel 1 and U-PLEX Chemokine Combo immunoassays (Meso Scale Discovery, Maryland, USA) were used to determine cytokine concentrations in conditioned media from cell lines or mouse serum. Samples were analyzed according to the manufacturer's recommendations.

Oil Red O staining of descending aorta
Oil Red O (0.2 g, Sigma) was dissolved in 100 ml of isopropanol and filtered through Whatman No. 1 filter paper (stock solution). The working stain was prepared by diluting the stock solution with distilled water (6:4). Adventitial tissue was carefully removed from the descending aorta, the artery opened along the longitudinal axis, rinsed in distilled water, rinsed in 60 % isopropanol for 30 sec, stained with Oil Red O working stain for 15 min, rinsed again in isopropanol and returned to distilled water. The vessel was laid flat on a glass slide, mounted in water, covered with a coverslip and imaged *en face* using an Olympus BX51 microscope equipped with an Olympus Lumenera Infinity 3 digital camera and analyzed using ImageJ software. The percentage of the luminal surface area stained by Oil Red O was determined.

Histological Analysis
Atherosclerosis extent and composition was analyzed as described previously[9]. Briefly, sections from the aortic root were stained with Haematoxylin and Eosin (H&E), Masson's,

8oxoG and TUNEL. Plaque extent and composition was measured using ImageJ analysis software with an observer blind to experimental group. SMA content of fibrous caps was defined using Masson's Trichrome to identify the fibrous cap region. Fibrous caps were defined as the area rich in VSMCs and proteoglycan overlying the cholesterol-rich, matrix-poor, acellular regions of the necrotic cores. Very small plaques (<90,000μm^2) were not included in the above analysis since it was impossible to demarcate a fibrous cap.

Cell culture
Human, rat and mouse VSMCs were cultured in Dulbecco's modified Eagle's medium (DMEM, Sigma) supplemented with 100 U/ml penicillin, 100 μg/ml streptomycin, 2 mM L-glutamine, and 10 % FCS.

Human VSMCs were cultured from explants. The endothelial layer was removed using a scalpel and tissue cut into 2-3mm² pieces, placed into 6-well plates containing 1ml media and grown for 1-2 weeks to allow cells to emerge. Human VSMCs were studied at passages 2–5; VSMC cultures from individual patients were not pooled. Rat and mouse aortic VSMCs were prepared by removing surrounding tissues and enzymatic dispersion using Type I collagenase (1 mg/ml, Sigma) and elastase (0.5 mg/ml, Worthington Biochemical) in serum-free medium for 1h at 37 °C.

Cells were treated with tert-butyl hydrogen peroxide (t-BHP, Sigma), C646 (Sigma), CTPB (Sigma), cycloheximide (Sigma) or MG132 (Sigma) as indicated in figure legends.

Transfections and virus infections
Retrovirus infection was used to produce stable expression of wild type OGG1 or the acetylation mutant OGG1$^{K\text{-}R}$ in rat aortic VSMCs. hOGG1 was subcloned from pCMV-myc-Nuc-hOGG1 (Addgene) and site-directed mutagenesis performed using the QuikChange Lightning Multi Site-Directed Mutagenesis Kit (Stratagene) following the manufacturer's instructions.

pBabe-puro vectors encoding human OGG1 or OGG1$^{K\text{-}R}$ were used to transfect Phoenix™ packaging cells (Orbigen) using SuperFect (Qiagen). Virus-producing cells were selected with hygromycin B (300 μg/ml; Calbiochem). VSMCs were infected with the virus suspension in the presence of 8 μg/ml Polybrene (hexadimethrine bromide, Sigma), and selected with 5 μg/ml of puromycin (Sigma).

CRISPR-mediated gene silencing
For gene silencing experiments, rat aortic VSMCs were transfected with a U6-gRNA/CMV-Cas9-GFP CRISPR plasmid targeting OGG1 (Sigma). To generate rat OGG1$^{-/-}$ VSMCs, cells were trypsinized 3 days after transfection and resuspended in PBS with 0.1% BSA and 2 mM EDTA. Single GFP-positive cells were sorted by FACS into 96-well plates, and clonal cell populations expanded. OGG1 protein expression of each clone was examined by immunoblotting, and clones with efficient OGG1 knockout used for further studies. Genomic DNA was extracted from 1×10^6 cells, and locus-specific cleavage was analyzed using the GeneArt Genomic Cleavage Detection Kit (Life Technologies).

Real-time polymerase chain reaction
RNA extraction from mouse tissues and primary VSMCs was performed using the RNeasy kit (Qiagen). First strand cDNA synthesis was performed using Superscript III Reverse Transcriptase (Invitrogen). Quantitative real-time PCR was performed using Rotor-Gene SYBR Green PCR Kit (Qiagen) on a Rotor-Gene 6000 QPCR thermocycler (Corbett Research). Oligonucleotide sequences used are listed in **Supplementary Table S1**.

Oligonucleotide incision assay

8oxoG BER activity in nuclear lysates was determined using a 40-mer oligonucleotide containing an 8oxoG at position 19 and labelled at the 3' end with indodicarbocyanine (5'-AGAGAAGAAGAAGAA(G*)AGATGGGTTATTCGAA-CTAGC-Cy5Sp-3'). This oligonucleotide was hybridized to its complementary sequence containing a cytosine opposite the 8oxoG lesion (G*). Nuclear extracts were added to a 10μL standard incision reaction mixture containing 200 fmoles of the 8oxoG:C-labeled duplex in 20 mmol/L Tris–HCl (pH 7.1), 1 mmol/L EDTA, 200 mmol/L NaCl, 1 mg/mL bovine serum albumin (BSA), and 5% glycerol. After 15 min at 37°C, the incision reaction was stopped by adding 4 μL of formamide dye and heating for 5 min at 95°C. The cleaved product was separated from the intact substrate in a 20% polyacrylamide gel containing 8 M urea in Tris-borate-EDTA buffer, pH 8.4. Fluorescence in the separated DNA bands was visualized using a LI-COR Odyssey CLx system.

8oxoG ELISA
Cell lysates were assessed for 8oxoG expression using a competitive ELISA assay (Abcam) as per the manufacturer's instructions. Total DNA was purified from VSMC lysates by using the DNeasy Blood & Tissue Kit (Qiagen). DNA was digested using nuclease P1 following the manufacturer's instructions. The pH was adjusted to 7.5-8.5 using 1M Tris and 1 unit alkaline phosphatase added per 100 μg DNA and incubated at 37°C for 30 minutes. Samples were boiled for 10 min and placed on ice until use. Absorbance was measured on a Synergy HT Plate Reader (Biotek) with standard curves generated from known concentrations of 8oxoG standards.

Intracellular ROS measurement
Intracellular ROS was measured by using the oxidant-sensitive fluorescent probe 5-(and-6)-chloromethyl-2',7'-dichlorodihydrofluorescein diacetate acetyl ester (CM-H2DCFDA,10 mM; Molecular Probes). Cells were incubated continuously with CM-H2DCFDA during experimental conditions. Subsequently, cells were washed and lysed in 0.1% Triton X-100. Fluorescence was measured in the Biotek synergy HT plate reader with excitation of 488 nm and emission of 530 nm.

Annexin V/PI flow cytometry
Cell death was determined using an Apoptosis Detection kit according to the manufacturer's instructions (BD BioSciences). Briefly, cells (1 × 10^5 cells/sample) were washed twice in cold PBS and suspended in binding buffer containing fluorescein isothiocyanate-conjugated annexin V (10 μg/ml) and PI (10 μg/ml). The cell suspension was incubated in the dark for 15 min and fluorescence measured using an Accuri C6 flow cytometer (BD Biosciences). A total of 10,000 events were analyzed for each sample with Cell Quest software (BD Biosciences).

Comet assay
5 × 10^4 VSMCs were washed with PBS, resuspended in 150 μl 1% low melting point agarose (LMPA) and left to cool on Gel-bond films (Trevigen) at 4˚C for 10 min to set the agarose. Gel-bond films were incubated overnight in lysis buffer at 4˚C, washed briefly with water, and placed in alkaline electrophoresis buffer for 30 min prior to electrophoresis at 26V for 30 min at 4˚C. Gel-bond films were washed in neutralization buffer (100 mM Tris pH 7.6) and subsequently immersed in ice-cold ethanol for 10 min. Films were dried overnight, rehydrated in distilled H_2O for 10 min and stained with 2μg/ml ethidium bromide (Sigma) solution in the dark at RT for 2 h. Multiple images per slide were analysed using a Zeiss microscope and comet tail length/moment measured using Comet Assay IV software (Perception Instruments).

Immunofluorescence
VSMCs for immunofluorescence and Proximity Ligation Assay (PLA) were cultured in chamber slides (ThermoFisher Scientific), fixed with 4 % paraformaldehyde for 15 min and

permeabilized for 5 min with 0.5% Triton X-100 in PBS prior to blocking with 3% BSA for 1 h. For immunofluorescence, cells were incubated with the following primary antibodies: rabbit anti-acetyl OGG1 (1:200, ab93670, Abcam) and rabbit anti-myc tag (1;1000, 2272S, Cell Signaling). Secondary antibodies were anti-rabbit Alexa Fluor 568, anti-mouse Alexa Fluor 568 and anti-mouse Alexa Fluor 594 (1:1000, Invitrogen). Nuclei were visualized using DAPI. PLA was performed according to manufacturer's instructions using the Duolink® In Situ Red Kit Mouse/Rabbit (Sigma) and the following primary antibodies: rabbit-anti OGG1 (1:200, PA1-16505, Thermofisher), mouse anti-p300 (1:100, NB100-616, Novus) and mouse anti-SIRT1 (1:100, 8469S, Cell Signaling). Nuclei were visualized using DAPI. Slides were mounted with DAPI-containing medium (Sigma or Vectashield, Vector Labs) and visualized with a BX51 fluorescent microscope (Olympus).

Immunoprecipitation
VSMCs were harvested in RIPA lysis buffer (20 mM Tris-HCl pH7.5, 150 mM NaCl and Triton 0.5 %) supplemented with protease and phosphatase inhibitor cocktails (Sigma). After clarification by centrifugation, 800 µg of total protein cell lysate was incubated with 4 µg of rabbit anti-OGG1 antibody (PA1-16505, Thermofisher) overnight at 4 °C. Immuno-complexes were precipitated with Pierce protein A/G magnetic beads for 4h at 4°C and detected by western blot with goat anti-OGG1 (PA5-18747, 1:500, Thermofisher), mouse anti-p300 (RW128, 1:500, Millipore), or mouse anti-SIRT1 (8469S, 1:1000, Cell Signaling) antibodies. Primary antibodies were detected using HRP-conjugated secondary antibodies and chemiluminescence (Amersham ECL or ECL prime reagents) detection.

Western blotting
Whole cell protein lysates were prepared using CelLytic reagent (Sigma). Immunoblotting of cell lysates was performed according to standard conditions. Immunoblots were labelled with the following primary antibodies: rabbit anti-acetyl histone 4 (1:1000, 06-866, Millipore), rabbit anti-acetyl OGG1 (1:1000, ab93670, Abcam), rabbit anti-myc tag (1;1000, 2272S, Cell Signalling, rabbit anti-NEIL1 (1:1000, ab128294, Abcam), mouse anti-NTH1 (1:500, ab70726, Abcam), rabbit anti-OGG1 (1:1000, ab124741, Abcam), mouse anti-p300 (1:500, RW128, Millipore), mouse anti-SIRT1 (1:1000, 8469S, Cell Signaling). Primary antibodies were detected using fluorescently labelled secondary antibodies: goat anti-rabbit IgG DyLight 680 and goat anti-mouse IgG Dylight 800 (Thermo Scientific). Detection and quantification of fluorescence intensity were performed using an Odyssey® CLx imaging system (LI-COR Biosciences, Lincoln) and Odyssey® 2.1 software. In some instances, HRP-conjugated secondary antibodies were used for chemiluminescence detection and protein levels quantified by densitometry and normalized against loading controls.

ChIP-qPCR
ChIP was performed using the ChIP-IT express kit (Active Motif), VSMCs were crosslinked for 10 min with formaldehyde (to a final concentration of 1 %). Chromatin was sheared using a Bioruptor UCD-200 ultrasound sonicator (Diagenode), resulting in DNA fragments of 500–1000 bp in size. Chromatin was immunoprecipitated with 2 µg mouse anti-8oxoG antibody (MAB3560, Millipore) or negative control mouse IgG (PP64, Millipore). Immunoprecipitated DNA was then used as a template for quantitative PCR using primers specific for genomic loci. Oligonucleotide sequences used are listed in Supplementary Table S1.

Supplemental Tables

Primers		Oligonucleotide Sequences
CASP1 (mouse)	Forward	5'- GCCCACCACTGAAAGAGTGA -3'
	Reverse	5'- TCTTCACTTCCTGCCCACAG-3'
CCL2 (mouse)	Forward	5'- AGCTGTAGTTTTTGTCACCAAGC -3'
	Reverse	5'- GTGCTGAAGACCTTAGGGCA -3'
GAPDH (human)	Forward	5'- GATGCCCCCATGTTCGTCAT-3'
	Reverse	5'- TGCAGGAGGCATTGCTGATG-3'
GAPDH (mouse)	Forward	5'- GGGTCCCAGCTTAGGTTCATC-3'
	Reverse	5'- CCCAATACGGCCAAATCCGT-3'
HPRT (mouse)	Forward	5'- ACAGGCCAGACTTTGTTGGA-3'
	Reverse	5'- TGCAGATTCAACTTGCGCTC-3'
NLRP1 (mouse)	Forward	5'- TGGTTCAGGGATGCTGGAAA-3'
	Reverse	5'- GCTGCTGGGCACTAGTATCTC-3'
NLRP3 (mouse)	Forward	5'- TCCTGGCTGTAACATTCGGAG-3'
	Reverse	5'- TGCAAGATCCTGACAACATGC-3'
Il1b (mouse)	Forward	5'- GCCACCTTTTGACAGTGATGAG-3'
	Reverse	5'- GACAGCCCAGGTCAAAGGTT-3'
IL6 (mouse)	Forward	5'- GACAAAGCCAGAGTCCTTCAGA-3'
	Reverse	5'- TGTGACTCCAGCTTATCTCTTGG -3'
IL18 (mouse)	Forward	5'- TCTTGGCCCAGGAACAATGG-3'
	Reverse	5'- ACAGTGAAGTCGGCCAAAGT-3'
OGG1 human	Forward	5'- GACTACAGCTGGCACCCTAC-3'
	Reverse	5'- CACTGAACAGCACCGCTTG-3'
OGG1 (genotyping mouse)	Forward	5'- GAGACACATCTTGCAGGGAGC-3'
	Reverse	5'- ACTCAGGACCTTCAGAAGAGCA-3'
	Neo	5'- GATGGATACTTTCTCGCAGG-3'
OGG1 (mouse)	Forward	5'- CCACCCTAGAGGAGCTGGAA-3'
	Reverse	5'- CAGCAGTCTCACACCTTGGA-3'
P300 (human)	Forward	5'- GCAGTGTGCCAAACCAGATG-3'
	Reverse	5'- CATAGCCCATAGGCGGGTTG-3'
SIRT1 (human)	Forward	5'- GGGCTGCGGTTCCTACTG-3'
	Reverse	5'- CAGACACCTATCCGTGGCCT-3'
Telomere ChIP	Forward	5'- GGTTTTTGAGGGTGAGGGTGAGGGTGAGGGTGAGGGT-3'
	Reverse	5'- TCCCGACTATCCCTATCCCTATCCCTATCCCTATCCCTA-3'
TNFα (mouse)	Forward	5'- GATCGGTCCCCAAAGGGATG -3'
	Reverse	5'- CCACTTGGTGGTTTGTGAGTG-3'

Supplemental Table I Oligonucleotides used in this study

Supplemental Figures and Figure Legends

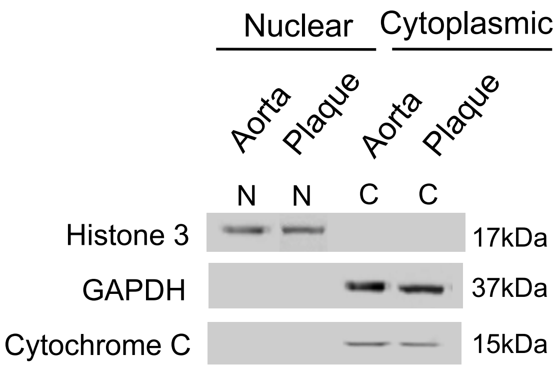

Supplemental Figure I
Western blot analysis for the nuclear marker histone H3, cytoplasmic marker GAPDH, and mitochondrial marker cytochrome c in nuclear or cytoplasmic subfractions of human plaque or normal aortic VSMCs (*n*=3).

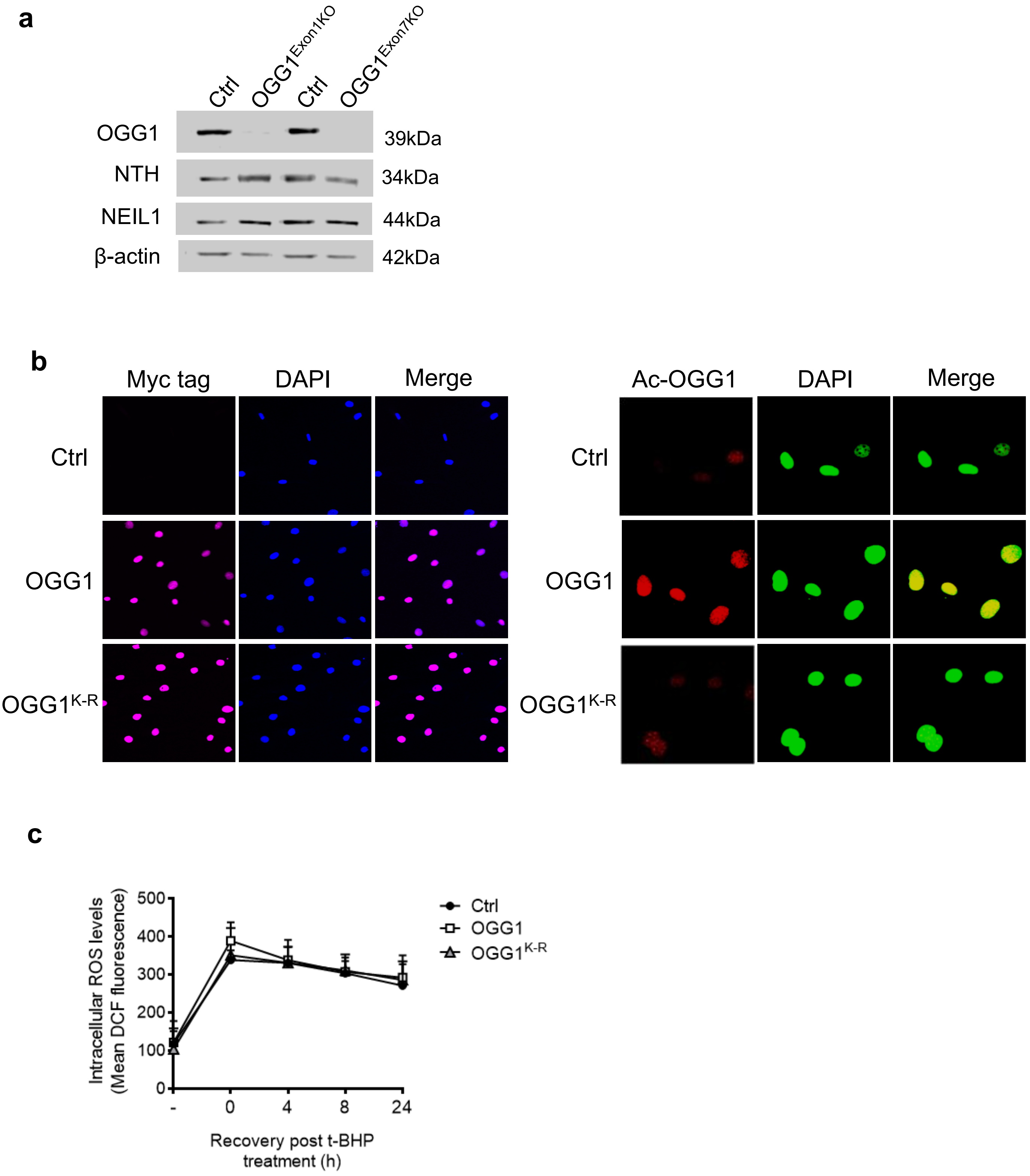

Supplemental Figure II

(**a**) Western blot analysis for OGG1, NEIL1 and NTH1 in Control (Ctrl), $OGG1^{Exon1KO}$ and $OGG1^{Exon7KO}$ rat VSMCs (n=4). (**b**) Immunofluorescence analysis of transgene expression (myc tag; left panel) or Ac-OGG1 (right panel) in VSMCs expressing the empty vector (Ctrl), OGG1 or $OGG1^{K\text{-}R}$ (n=3). (**c**) Quantification of intracellular ROS levels in Ctrl, OGG1 or $OGG1^{K\text{-}R}$ VSMCs measured by DCFDA fluorescence (n=3).

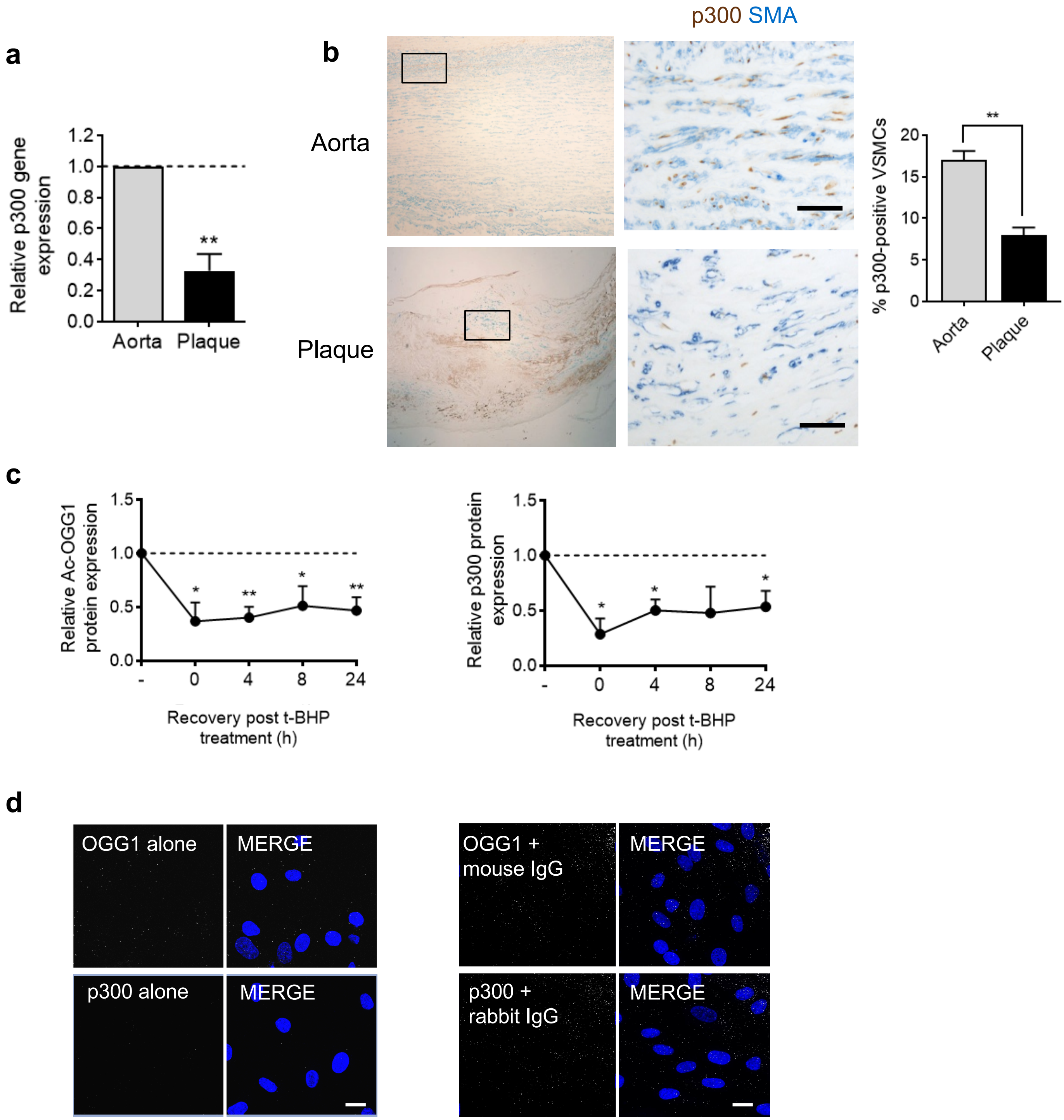

Supplemental Figure III

(a) qPCR analysis of p300 expression in cultured human plaque or normal aortic VSMCs (*n*=4). **(b)** Immunohistochemistry analysis and quantification of p300 (brown) and αSMA (blue) staining in tissue sections of human plaques and normal aorta (*n*=10). High power magnification views of areas outlined on left are shown and quantification. Scale markers, 25μm. **(c)** Quantification of relative Ac-OGG1 and p300 protein expression after t-BHP treatment and 0-24h recovery in control VSMCs (*n*=3). **(d)** Negative control PLA analysis using rabbit anti-OGG1 and mouse anti-p300 antibodies alone or with mouse IgG and rabbit IgG respectively (*n*=3). All graphical data are mean ± SEM, *P< 0.05, **P< 0.01, Student's t-test.

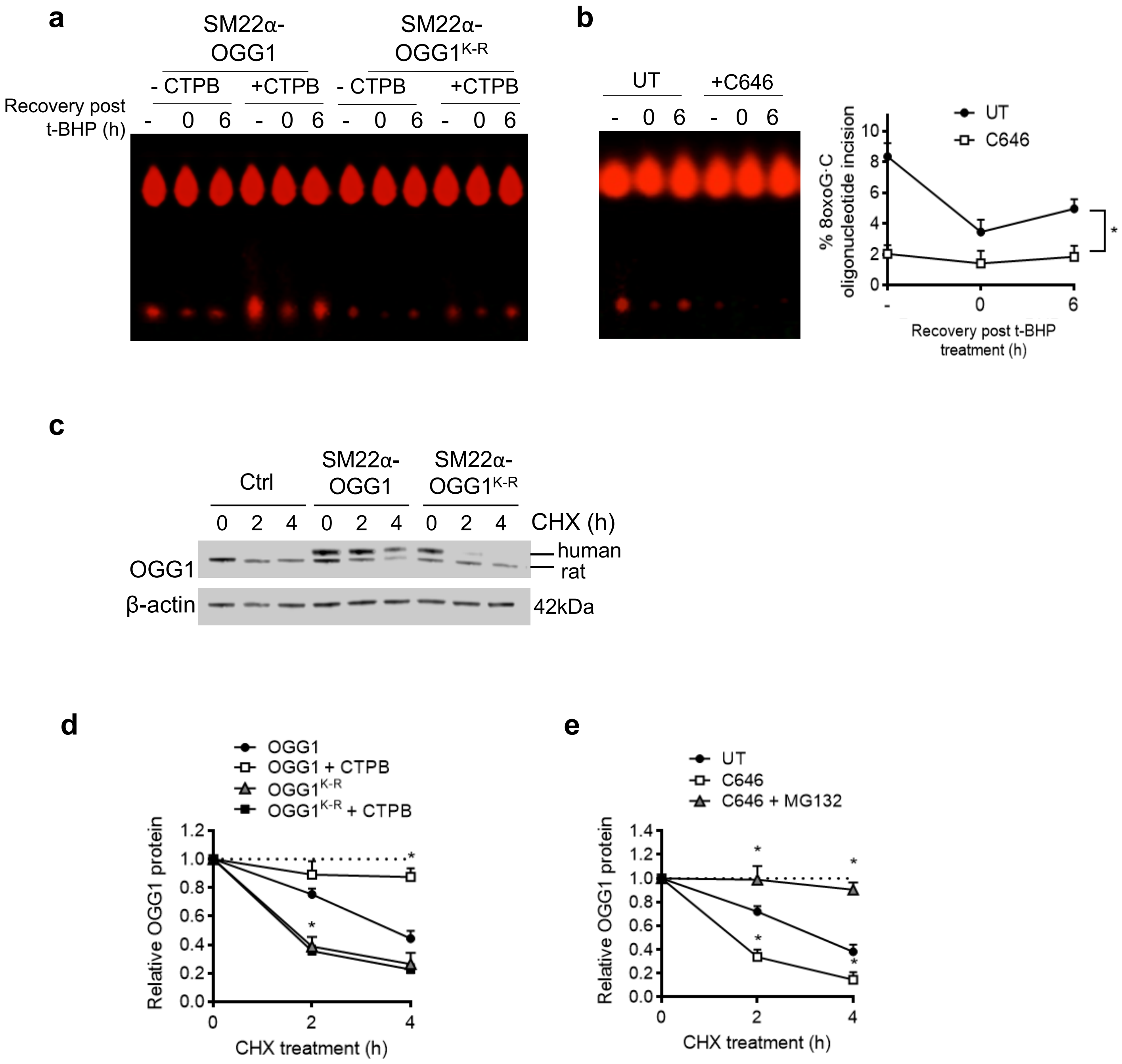

Supplemental Figure IV

(a) Representative BER assay in SM22α-OGG1 and SM22α-OGG1^{K-R} mouse VSMCs (left) ±CTPB (10μM) after 1h t-BHP treatment and 0- 6h recovery (*n*=3). **(b)** BER assay and quantification in control VSMCs either untreated (UT, -), after 1h t-BHP (0) or 6h recovery ± C646 (10μM) (*n*=3). **(c)** Western blot showing levels of OGG1 after CHX treatment in Ctrl, OGG1 or OGG1^{K-R} VSMCs (*n*=4). **(d)** Quantification of Western blot in Figure 3g showing levels of OGG1 after CHX treatment in OGG1 or OGG1^{K-R} VSMCs ±CTPB (*n*=3). **(e)** Quantification of Western blot in Figure 3h of OGG1 levels after CHX treatment in control VSMCs ± p300 inhibitor C646 and MG132 (10μM) (*n*=3). All graphical data are mean ± SEM, *P< 0.05, Student's t-test or one-way ANOVA (Bonferroni post hoc).

a

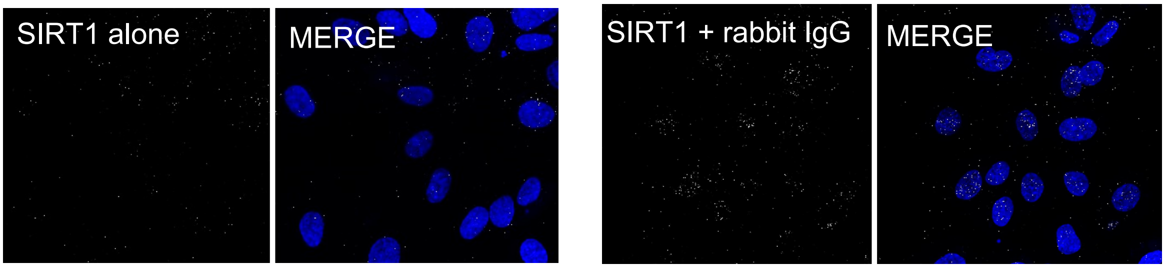

b

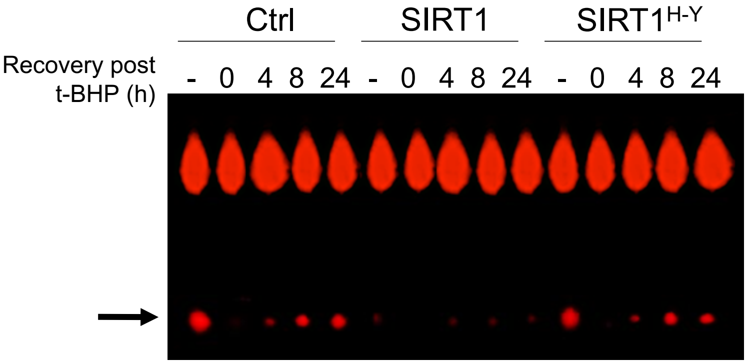

c

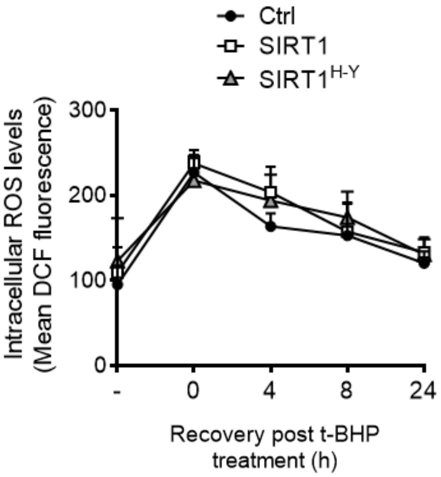

Supplemental Figure V

(a) PLA analysis using mouse anti-SIRT1 antibody alone or with rabbit IgG as negative controls (*n*=3). **(b)** Representative image of BER assay gel showing Ctrl, SIRT1 and SIRT1$^{H\text{-}Y}$ cells after 1h t-BHP treatment and 0-24h recovery (*n*=3). **(c)** Quantification of intracellular ROS levels in Ctrl, SIRT1 and SIRT1$^{H\text{-}Y}$ VSMCs after 1h t-BHP treatment and 0-24h recovery measured by DCFDA fluorescence (n=3).

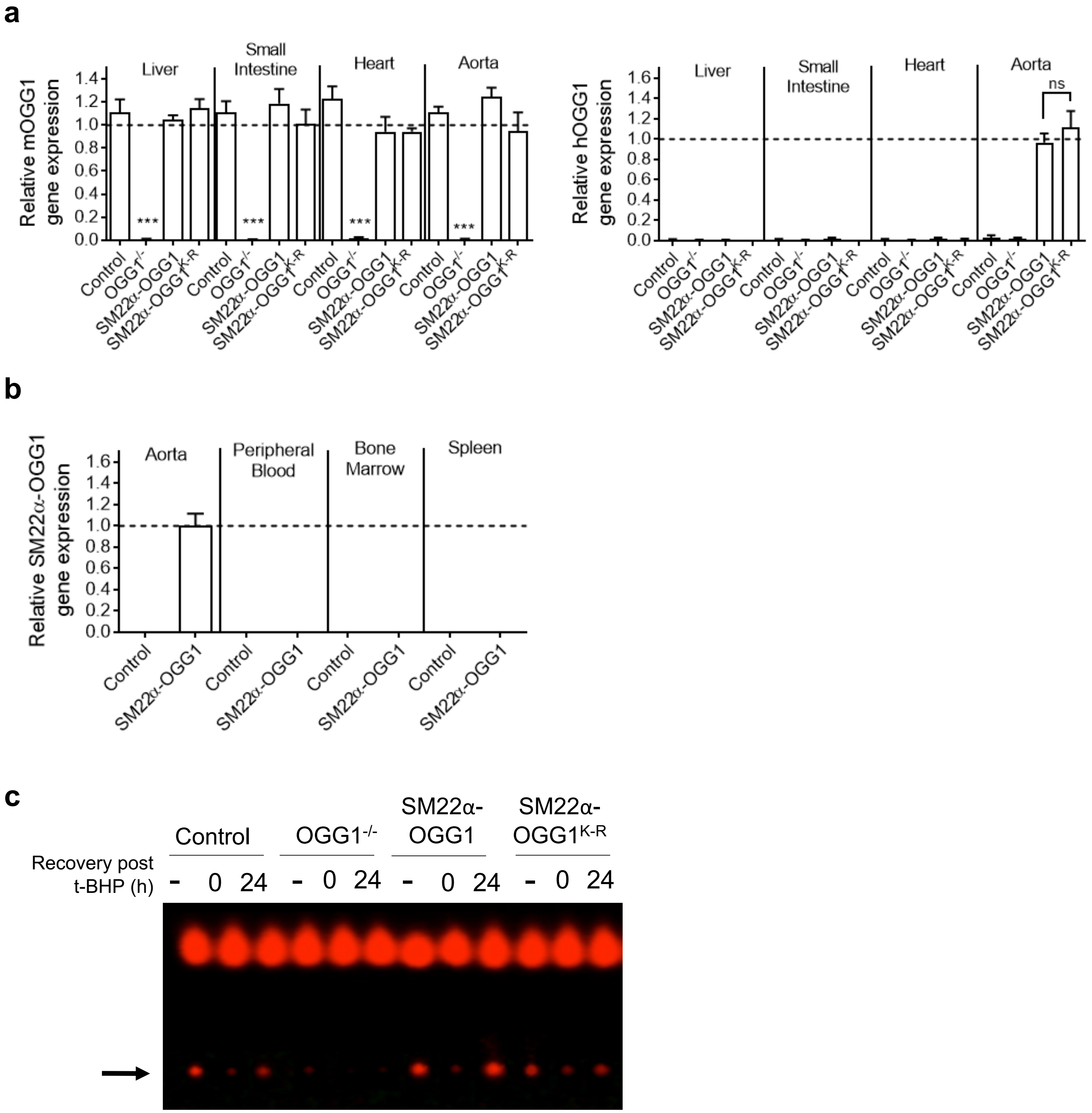

Supplemental Figure VI

(a) qPCR analysis of mouse (mOGG1; left panel) and human (hOGG1; right panel) OGG1 in liver, small intestine, heart and aorta tissue from control, SM22α-OGG1,SM22α-OGG1^{K-R} and OGG1$^{-/-}$ mice (*n*=4). **(b)** qPCR analysis of SM22α-OGG1 in aorta tissue, peripheral blood, bone marrow cells and spleen from control and SM22α-OGG1 mice (*n*=3). **(c)** Representative BER assay in Control, SM22α-OGG1,SM22α-OGG1^{K-R} and OGG1$^{-/-}$ mouse VSMCs after 1h t-BHP treatment and 0-24h recovery (*n*=3). All graphical data are mean ± SEM, ***P< 0.001, one-way ANOVA (Bonferroni post hoc).

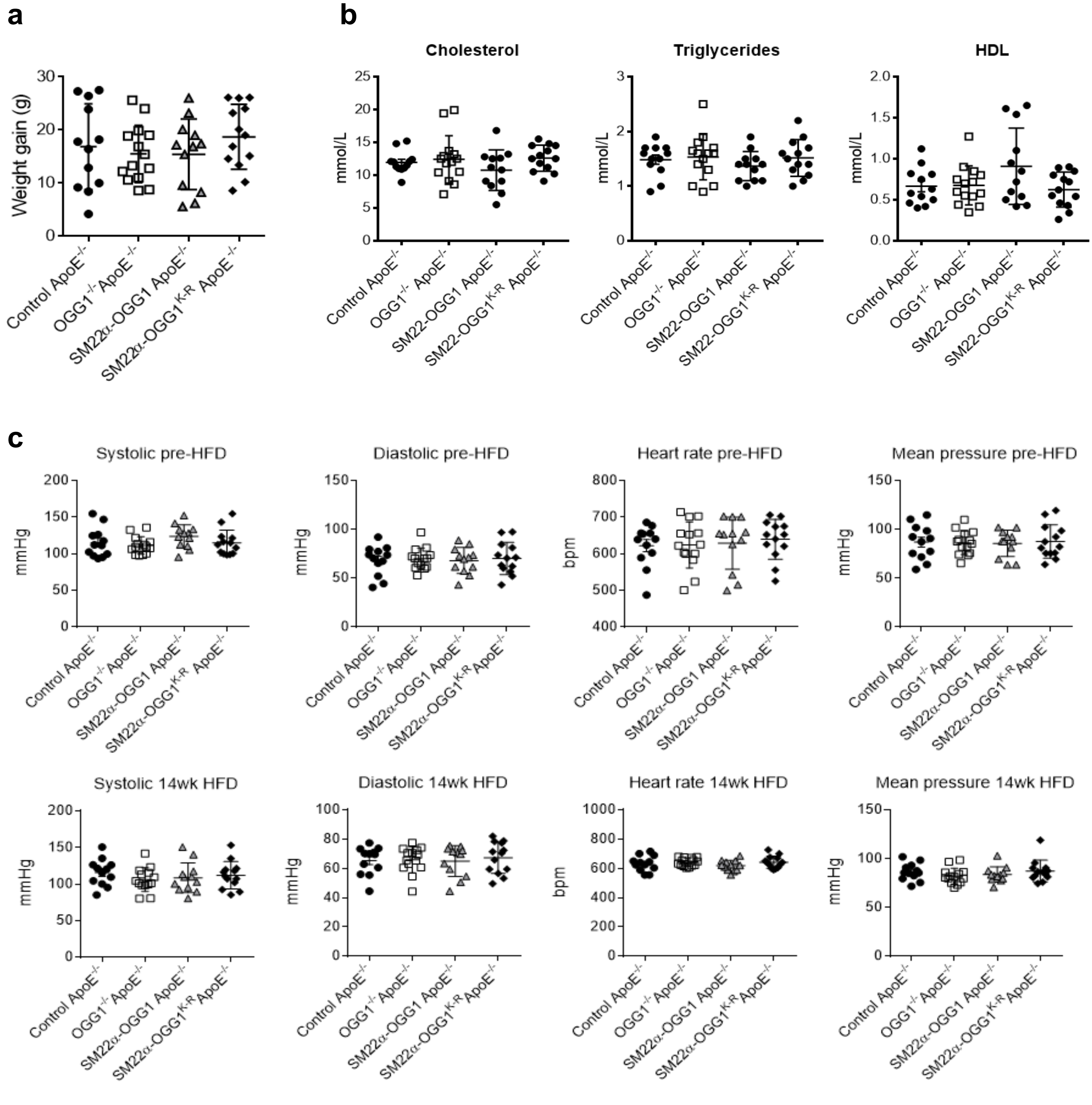

Supplemental Figure VII

(a) Body weight gain, **(b)** serum lipids and **(c)** blood pressure and heart rates in Control ApoE$^{-/-}$ (*n*=12), OGG1$^{-/-}$ ApoE$^{-/-}$ (*n*=14), SM22α-OGG1 ApoE$^{-/-}$ (*n*=12) and SM22α-OGG1^{K-R} ApoE$^{-/-}$ (*n*=13) mice pre- and post- fat feeding from 8-22 weeks (14w HFD).

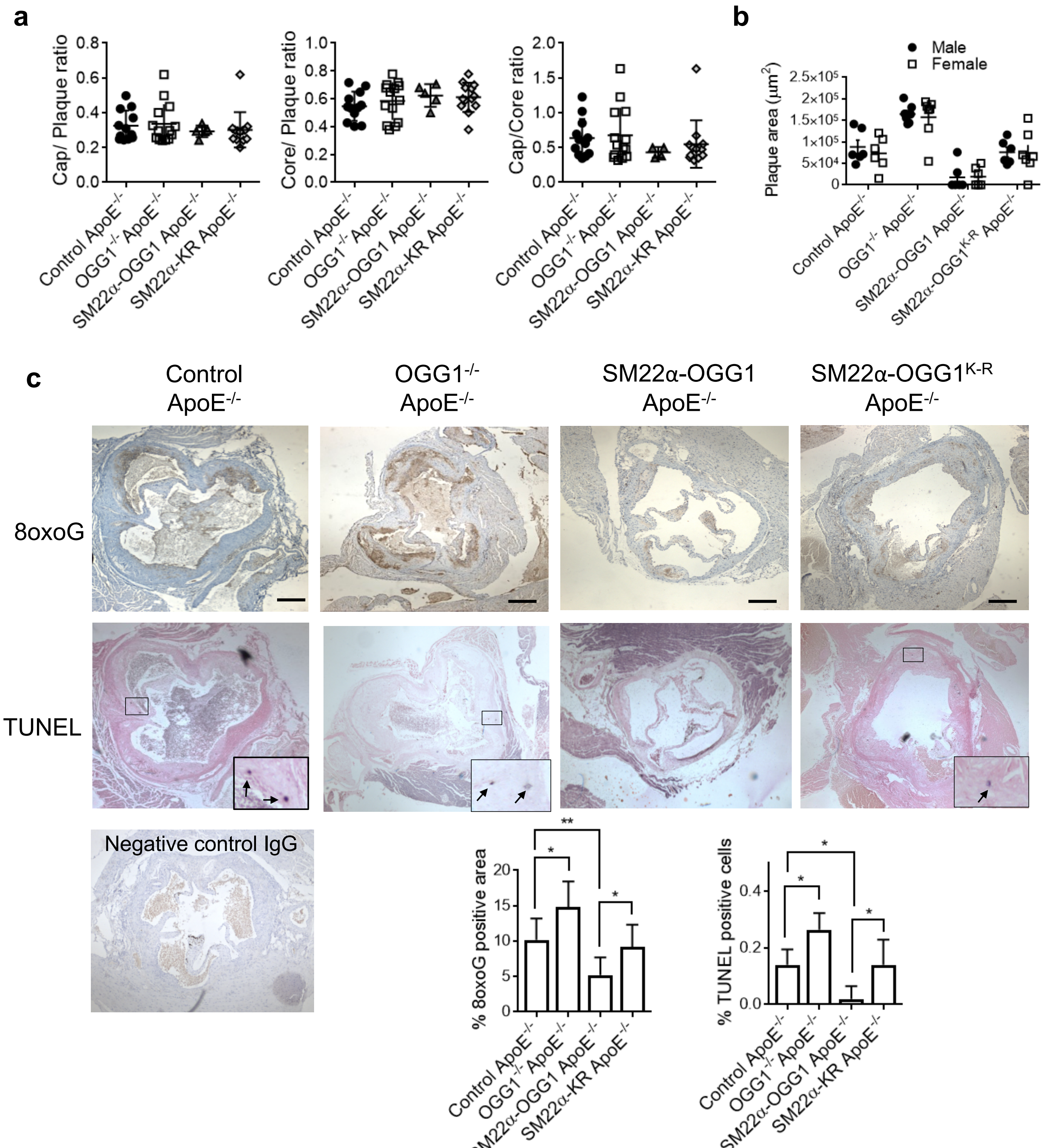

Supplemental Figure VIII

(a) Morphometry of plaques in experimental mice. Quantification of aortic root plaque, core and cap area ratios in control ApoE$^{-/-}$ (n=*12*), OGG1$^{-/-}$/ApoE$^{-/-}$ (n=*14*), SM22α-OGG1/ApoE$^{-/-}$ (n=*5* as caps/cores not discernible in very small lesions) and SM22α-OGG1^{K-R}/ApoE$^{-/-}$(n=*13*) mice at 22 weeks after fat feeding from 8 to 22 weeks (n= 11-14). **(b)** Plaque area of aortic roots in male and female study mice (μm^2). **(c)** 8oxoG and TUNEL immunohistochemistry analysis of Control ApoE$^{-/-}$ (*n*=12), OGG1$^{-/-}$ ApoE-/- (*n*=14), SM22α-OGG1 ApoE$^{-/-}$ (*n*=12) and SM22α-OGG1^{K-R} ApoE$^{-/-}$ (*n*=13) mouse aortic roots at 22 weeks after fat feeding. Negative controls and % 8oxoG-positive aortic area and % TUNEL-positive aortic cells in aortic roots are shown below. All graphical data are mean ± SEM, *P< 0.05, **P<0.01, one-way ANOVA (Bonferroni post hoc).

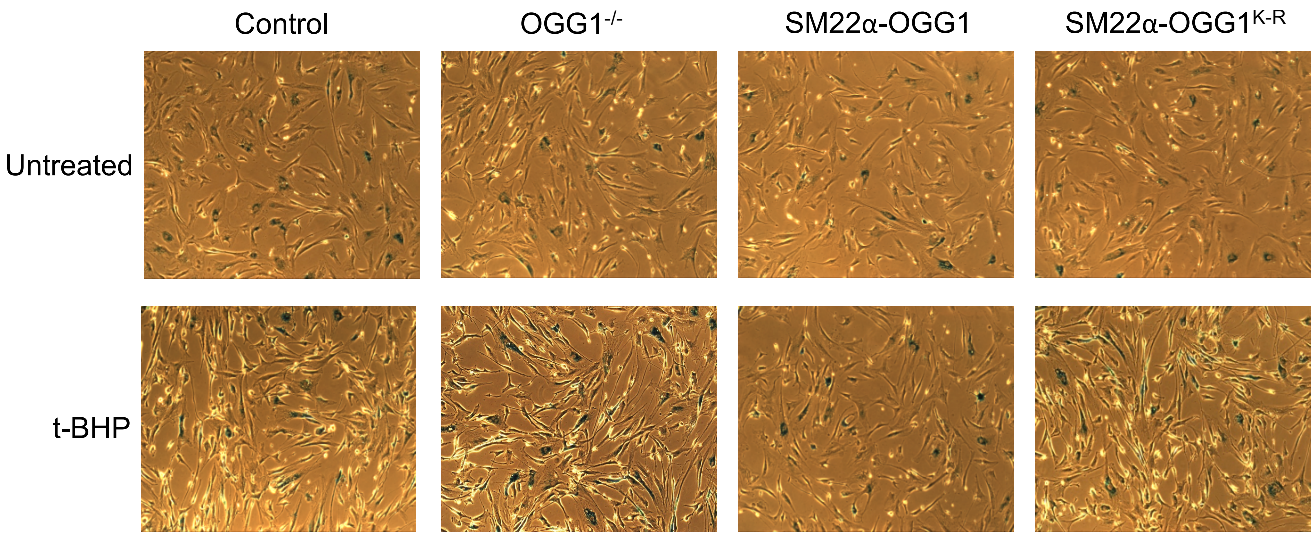

Supplemental Figure IX
Representative images of senescence-associated β-galactosidase activity of VSMCs cultured from Control, OGG1$^{-/-}$, SM22α-OGG1, SM22α-OGG1$^{K\text{-}R}$ mice (n=3).

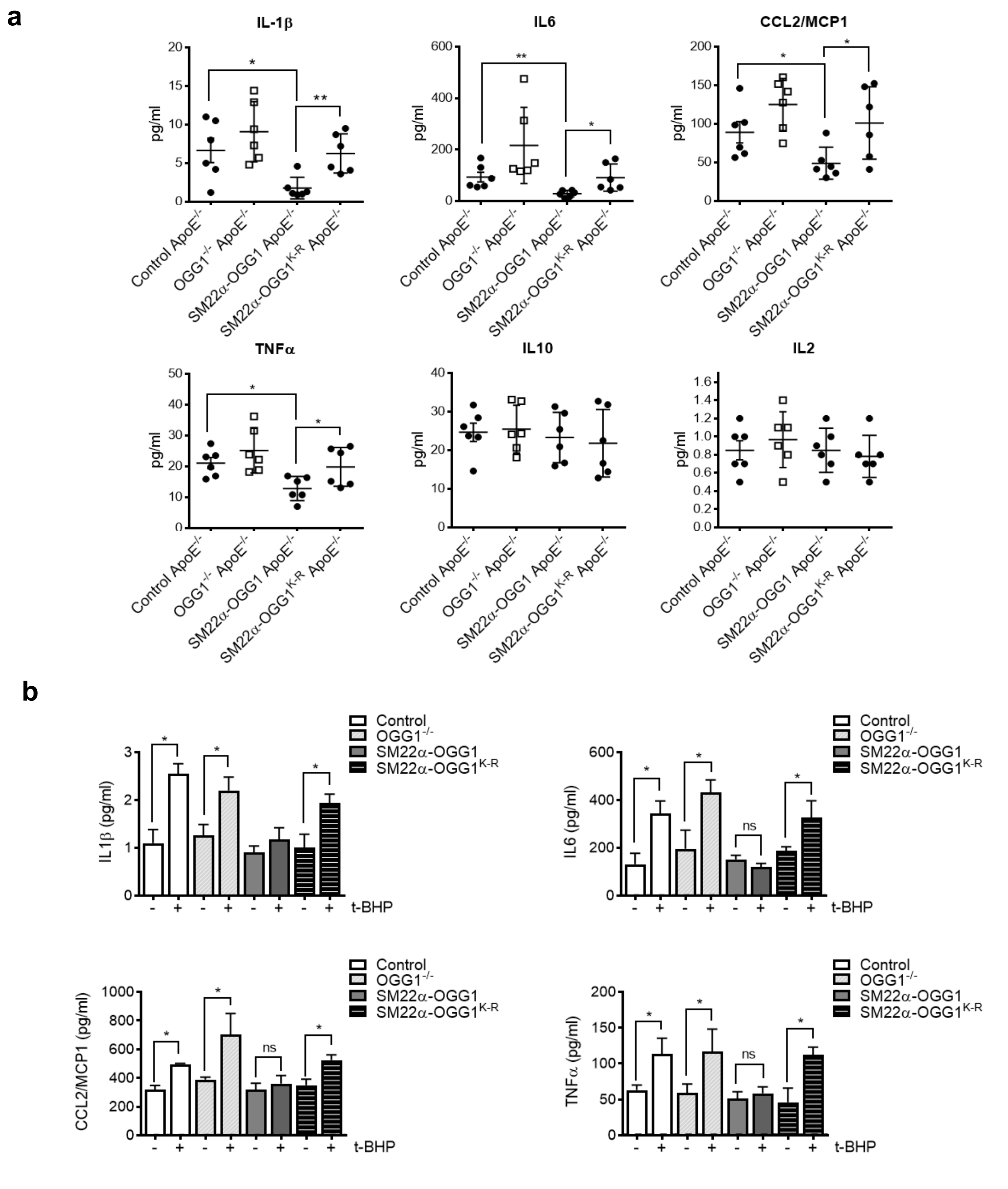

Supplemental Figure X

(a) Serum cytokines from a subset of Control, OGG1$^{-/-}$, SM22α-OGG1, SM22α-OGG1$^{K\text{-}R}$ mice at 22 weeks after fat feeding from 8-22 weeks (n=*6*). **(b)** Levels of CCL2/MCP1, IL1β, IL2, IL6, IL10 and TNFα in conditioned media from experimental mouse VSMC cultures stimulated with t-BHP measured by multiplex ELISA (n=3), Student's t-test. All graphical data are mean ± SEM, *P < 0.05, **P < 0.01, one-way ANOVA (Bonferroni post hoc).
